# AI‐Directed 3D Printing of Hierarchical Polyurethane Foams

**DOI:** 10.1002/advs.202515122

**Published:** 2025-11-29

**Authors:** Dhanush Patil, Jie Tian, Kun Jiang, Clarissa Westover, Sri Vaishnavi Thummalapalli, Arunachalam Ramanathan, Arpita Shome, Natalie Crutchfield, M. Taylor Sobczak, Sean Lee, Hongyue Sun, Elizabeth J. Brisbois, Hitesh Handa, Timothy E. Long, Xianqiao Wang, Kenan Song

**Affiliations:** ^1^ Mechanical Engineering, College of Engineering University of Georgia 302 E. Campus Rd Athens GA 30602 USA; ^2^ School for the Engineering of Matter, Transport, and Energy and Biodesign Center for Sustainable Macromolecular Materials and Manufacturing (SM3) Arizona State University Tempe AZ 85287 USA; ^3^ School of Chemical, Materials & Biomedical Engineering, College of Engineering University of Georgia Athens GA 30602 USA; ^4^ Department of Pharmaceutical & Biomedical Sciences, College of Pharmacy University of Georgia Athens GA 30602 USA; ^5^ School of Molecular Sciences and Biodesign Center for Sustainable Macromolecular Materials and Manufacturing (SM3) Arizona State University Tempe AZ 85287 USA; ^6^ Professor of Mechanical Engineering, School of Environmental, Civil, Agricultural and Mechanical (ECAM) University of Georgia Athens GA 30602 USA; ^7^ Associate Professor of Mechanical Engineering, School of Environmental, Civil, Agricultural and Mechanical (ECAM) and School of Chemical, Materials, and Biomedical Engineering (CMBE) University of Georgia Athens GA 30602 USA

**Keywords:** 3D printing, patterning, polyurethane foam, porous media

## Abstract

Hierarchical porous materials enable next‐generation protective, thermal, and biomedical devices by leveraging multiscale architectures with tunable mechanical and thermal properties. Current 3D printing techniques mainly yield periodic lattices and support limited polymer types, restricting patterning possibilities and scalability. Stochastic foam architectures, with heterogeneous and interconnected pores, mimic biological structures for improved energy dissipation and functional adaptability. However, scalable additive manufacturing of such foams remains scarcely explored. Here, a direct ink writing (DIW) strategy couples static mixer‐enabled reactive extrusion and in situ polymerization to manufacture stochastic polyurethane (PU) foams at ambient conditions, eliminating post‐processing. Precise control over pore size (0.2 µm to 1.2 mm), porosity (65–95%), and open‐cell architecture delivers thermal conductivities down to 0.067  W m^−1^ K^−1^ and elastic recovery exceeding 90% after 5000 cycles. A multi‐agent artificial intelligence framework enables the patterning of spatially organized, bioinspired motifs into print‐ready CAD geometries, resulting in architecturally structured foams with tailored anisotropy and spatial thermal management. Flow‐rate modulation further tunes morphology, optimizing the balance between stiffness and damping. This manufacturing platform integrates stochastic pore formation, AI‐guided patterning, and mechanical–thermal optimization to realize scalable, customizable materials for impact protection, wearable thermotherapy, and adaptive healthcare, advancing digital manufacturing, smart materials, and personalized function.

## Graphical abstract

Digitally guided direct ink writing, combined with AI‐generated design, enables the fabrication of hierarchical polyurethane foams with tunable multiscale porosity. This approach produces architected foams featuring interconnected open‐cell networks and tailored mechanical properties, advancing the development of adaptive, high‐performance materials for protective, structural, and wearable applications.

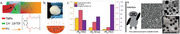

## 1 Introduction

Natural systems excel at organizing matter into hierarchical, multifunctional architectures to confer exceptional mechanical resilience and adaptive functionality, as exemplified by the graded porosity of bamboo, the energy‐dissipative peel of pomelo fruit, and the layered exoskeletons of arthropods.^[^ ^1^, ^2^, ^3^, ^4^, ^5^ ^]^ These structurally diverse and highly organized morphologies arise through intricate physio‐chemical processes, ultimately providing advantages such as superior impact resistance, thermal regulation, and structural lightweighting. Emulating such strategies, polymeric porous materials, most notably polyurethane (PU) foams have become indispensable across thermal insulation, cushioning, and protective applications, valued for their low density, chemical tunability, and processability.^[^ ^6^, ^7^, ^8^, ^9^ ^]^ However, engineered PU foams are typically constrained to simple, isotropic, microporous morphologies owing to the limitations of conventional batch foaming, where pore size and distribution are governed primarily by gas evolution dynamics during polymerization.^[^ ^10^, ^11^, ^12^ ^]^ This lack of control over pore spatial distribution, orientation, and hierarchical connectivity restricts the capacity of synthetic foams to emulate the exceptional combination of toughness, adaptability, and spatially resolved properties found in biological systems. As the demand for advanced foams that unite lightweight construction, programmable energy dissipation, and tailored thermal management grows particularly in fields such as personal protective equipment and wearable healthcare, recent advances in digital fabrication and machine learning offer a transformative route.^[^ ^13^, ^14^, ^15^ ^]^ These emerging tools enable the translation of nature's intricate architectural motifs into scalable, customizable PU foams, introducing unprecedented control over structural hierarchy, pore patterning, and multifunctional performance.

Despite the remarkable structural diversity achieved in nature through layer‐by‐layer assembly, as seen in bird beaks, pomelo peels, and plant vasculature, which achieve lightweight toughness, directional energy absorption, and multifunctionality through hierarchical design.^[^ ^16^, ^17^, ^18^ ^]^ Most current foam fabrication techniques, such as batch foaming, laser patterning, emulsion templating, and gas‐saturation methods, fail to replicate this complexity. These approaches lack macrostructural control, reproducibility, or scalability that may involve hazardous reagents or energy‐intensive post‐processing steps.^[^ ^19^, ^20^, ^21^, ^22^, ^23^ ^]^ Additive manufacturing (AM), such as direct ink writing (DIW), offers a transformative platform for creating porous materials with programmable geometry, tunable pore size distribution, and anisotropic mechanical behavior.^[^ ^24^, ^25^ ^]^ Unlike traditional batch foaming, which provides minimal control over pore formation, AM enables spatially resolved material placement, allowing fabrication of functionally graded and hierarchically structured architectures. However, advanced AM techniques, such as fused deposition modeling (FDM) combined with supercritical gas foaming, hydrogel‐based DIW, lattice printing via stereolithography (SLA) or digital light processing (DLP) 3D printing for lattice structures, remain constrained by slow production rates, limited material compatibility, reliance on specialized assemblies, and resins.^[^ ^26^, ^27^, ^28^, ^29^, ^30^ ^]^

Additionally, direct laser writing (DLW) via two‐photon polymerization enables ultrahigh spatial resolution and biomimetic architectures, while templated bubble writing offers fine control of pore nucleation. Nevertheless, both face significant challenges with scalability and throughput, largely restricting their use to small, specialized components.^[^ ^31^, ^32^ ^]^ These strategies are confined to periodic or highly ordered architectures, contrasting with the controlled randomness, chaotic yet functional, prevalent in natural materials. True “chaotic ordering” is fundamental to nature's optimized energy dissipation and adaptability, but has been inaccessible via conventional designs. These challenges underscore the urgent need for a scalable, customizable, and multi‐length scale fabrication strategy, such as one that seamlessly merges AI‐enabled digital design with continuous reactive extrusion to yield truly stochastic foams with interconnected hierarchical porosity, unlocking pathways to customizable, bioinspired materials for thermal management, impact mitigation, and wearable healthcare devices.

Integrating artificial intelligence (AI) and machine learning (ML) into these AM platforms further accelerates design iterations: large language models (LLM(s)), such as ChatGPT, can rapidly translate bioinspired prompts, e.g., lotus leaf venation, bird beak cores, or bamboo culm patterns, into digital geometries ready for fabrication.^[^ ^33^ ^]^ This capability opens new design spaces for multifunctional foams that bridge biological inspiration and engineered performance. For example, recent advances in dynamic covalent polymer networks have introduced recyclable and self‐healing thermoset materials that are compatible with high‐resolution 3D printing, enabling the creation of reprocessable printed structures for circular manufacturing.^[^ ^34^ ^]^ As another example, developments in chemical upcycling strategies have transformed commodity PU foams into high‐value 3D‐printable resins under mild conditions, expanding the material palette for hierarchical foam fabrication.^[^ ^35^ ^]^ However, translating AI‐generated stochastic architectures into functional printed foams remains nontrivial. The generative nature of LLMs can produce inconsistencies in pore connectivity and morphology, complicating manufacturability and print fidelity. Additionally, converting natural language into machine‐readable printing instructions introduces ambiguities that may lead to structural defects or performance deviations. Overcoming these challenges requires coupling AI‐driven creativity with physics‐informed validation and robust, continuous reactive extrusion processes.

This work introduces a novel hybrid strategy that integrates DIW with controlled in situ polymerization to fabricate stochastic PU foams featuring well‐defined hierarchical structure–property relationships. By leveraging a ChatGPT‐assisted multi‐agent framework and advanced text‐to‐image capabilities from LLMs, we have enabled rapid and iterative architectural design with precise modulation of spatial porosity gradients and stochastic microstructures. The printed foams feature multiscale, functionally graded architectures, combining macro‐scale porosity (ranging from 65% to 95%) for low‐velocity impact mitigation and micro‐scale pore sizes (0.2 µm to 1.2 mm) for enhanced viscoelastic energy dissipation. Our AI‐driven design and fabrication approach enables single‐step manufacturing under ambient conditions, circumventing the geometric limitations of conventional lattice‐based scaffolds and the uniformity constraints of bulk PU foams. Application‐specific demonstrations show elastic recovery exceeding 90% after 5000 tensile cycles and ultralow thermal conductivity as low as 0.067 W m^−1^ K^−1^, highlighting their potential for thermally insulating, energy‐absorbing systems. In particular, their high energy absorption and thermal regulation make them ideally suited for next‐generation helmet liners and protective gear, where localized impact damping and thermal comfort are critical. This multidisciplinary framework not only advances foam manufacturing through integrated material‐process‐design innovation but also highlights the potential of AI‐assisted engineering in medical devices and wearable safety equipment, establishing a scalable platform for producing adaptive, high‐performance porous materials.

## 2 Results and Discussion

### 2.1 Overview of AI‐Facilitated Biodesign and 3D Printing‐Enabled Rapid Prototyping

**Figure 1:**
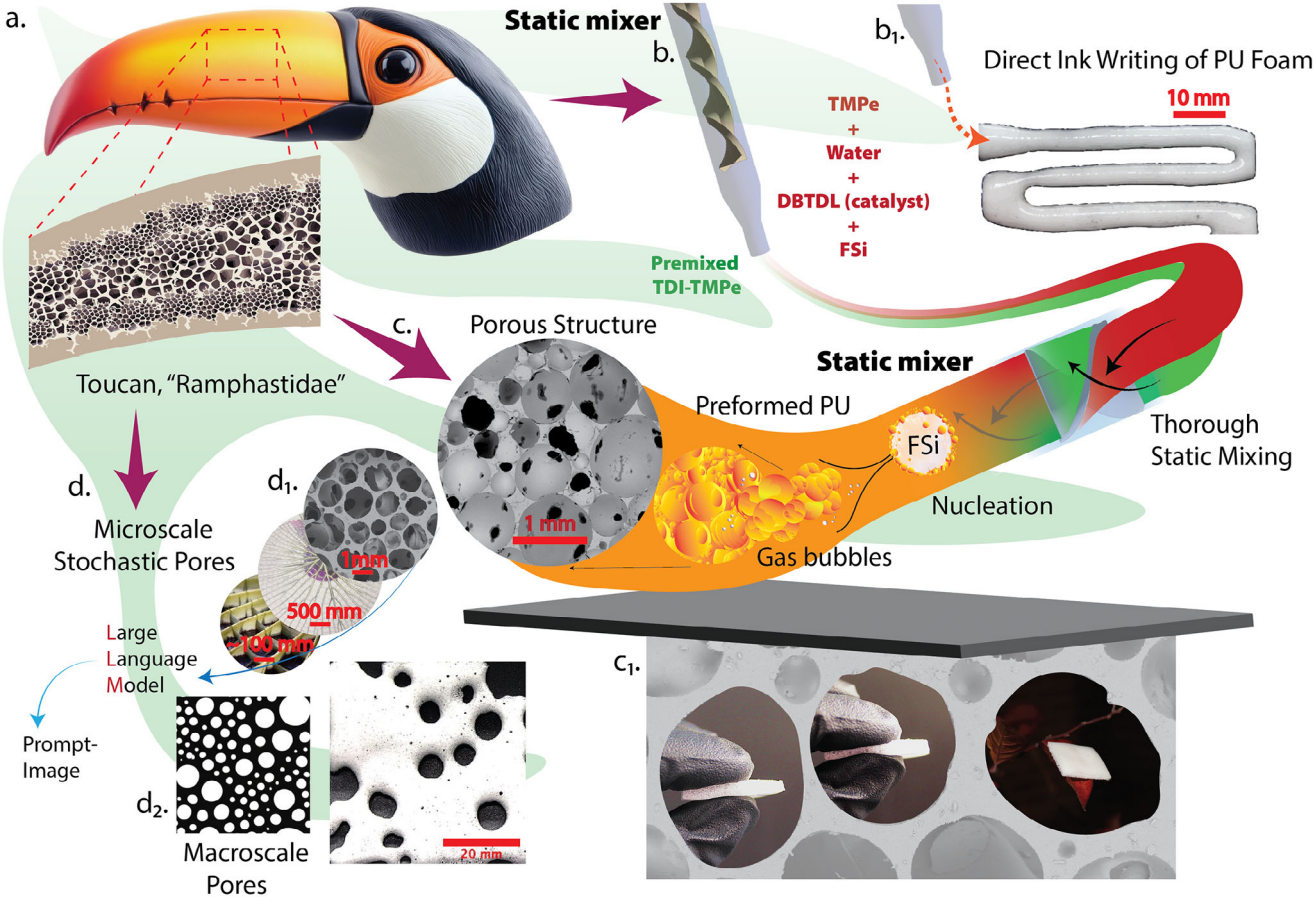
Schematic of AI‐assisted DIW of bioinspired PU foams containing non‐conventional, hierarchical porous architectures. a) The toucan beak* (Ramphastidae) provides structural inspiration due to its lightweight, hierarchical porous structure with exceptional energy absorption properties that guide the design of foam architecture (*Image licensed from Adobe Stock, stock.adobe.com). b) The DIW process involves dual‐barrel extrusion of TMPe, water, DBTDL catalyst, and fumed silica (FSi), premixed with TDI. (b_1_) The reactive components are homogenized via a static mixer, enabling nucleation and foam formation with the aid of high surface area FSi, producing printable PU foam. c) The resulting foamed structures display an interconnected porous morphology (SEM imaging). (c_1_) The PU foam demonstrates robust structural integrity under compression, enabled by in situ nucleation and static mixing. d) AI‐assisted workflows using LLMs generate prompt‐based porous designs. (d_1_) These support the creation of stochastic microscale pore geometries inspired by natural systems. (d_2_) Generated macroscale pore patterns (e.g., from prompt images) are realized through DIW to replicate bioinspired porosity at multiple scales.

This work presents a scalable and programmable approach to fabricate hierarchical porous PU foams by integrating DIW with real‐time in situ polymerization and AI‐driven design workflows. PU foams, traditionally employed in packaging, insulation, filtration, and mechanical damping, are valued for their tunable porosity and energy absorption properties.^[^ ^4^, ^5^, ^25^ ^]^ Recent advances have extended their utility to medical and safety domains, but conventional batch‐based methods based on urethane chemistry or lattice‐structured 3D printing from filaments limit the complexity and performance of foam architectures. Here, we bridge this gap by enabling continuous, real‐time fabrication of bioinspired, stochastic PU foams with tailored micro‐ and macro‐scale architectures. This innovation spanning bioinspired design, reactive extrusion, multiscale pore generation, and AI‐enabled prototyping is illustrated in **Figure** **1**.

The conceptual foundation of our design strategy originates from biological inspiration, specifically, the lightweight and energy‐dissipating structure of the toucan beak (Figure 1a). The toucan's beak exhibits a highly organized collagen core with stochastic porosity that balances strength, toughness, and compliance, serving as an effective blueprint for constructing multifunctional porous foams.^[^ ^17^, ^36^ ^]^ To replicate these features synthetically, we devised a DIW platform that utilizes static mixer‐enabled extrusion of reactive PU precursors, comprising toluene diisocyanate (TDI), trimethylolpropane ethoxylate (TMPe), water, dibutyltin dilaurate (DBTDL) catalyst, and fumed silica (FSi) nanoparticles (Figure 1b). These components are co‐injected through a dual‐barrel syringe system into a static mixer, where intensive shearing and rapid homogenization synchronize chemical crosslinking with gas bubble nucleation and dispersion.^[^ ^37^ ^]^ This simultaneous mixing led foaming process enables the formation of printable, rheology‐tuned PU inks with uniformly distributed gas domains, directly addressing the limitations of conventional batch foaming, which suffers from premature gelation.^[^ ^12^ ^]^

A critical advantage of this hybrid approach lies in its ability to simultaneously control polymerization kinetics and foam morphology during extrusion (Figure 1b_1_), also shown in **Table** **1** of the formulation details. The inclusion of high surface area of FSi particles enhances heterogeneous nucleation, resulting in a dense population of uniformly distributed microbubbles and enabling controlled in situ foaming under ambient conditions.^[^ ^38^ ^]^ This synchronized chemical–physical process yields a reactive ink with tunable viscoelasticity optimized for DIW, producing stable filaments that maintain geometric fidelity with minimal sagging or pore coalescence during deposition.^[^ ^39^ ^]^ As shown in Figure 1c, the printed PU foams form interconnected open‐cell microstructures with average cell sizes ranging from 0.2 µm to 1.2 mm and porosities between 65% and 95%. Such open‐cell structures are particularly advantageous for energy absorption and repeated loading conditions, as the interconnected struts and truss elements accommodate bending and buckling under stress, redistributing load without catastrophic fracture. By comparison, closed‐cell foams comprising isolated gas‐filled cavities offer higher compressive strength but limited recoverability upon deformation, making them less suited for cyclic impact scenarios.^[^ ^40^, ^41^ ^]^ The open‐cell morphology achieved here therefore provides a crucial balance between mechanical cushioning, structural stability, and lightweight performance. Microstructural fidelity is confirmed through SEM imaging (Figure 1c_1_), illustrating the uniform cellular distribution preserved post‐printing. Notably, this real‐time control over pore formation and polymer network stabilization eliminates post‐processing requirements and addresses long‐standing limitations of batch foaming and templated architectures, enabling reproducible, high‐resolution foam structures suitable for scalable applications.

**Table 1:** Comprehensive PU foam formulation and sample nomenclature.

Components	Std.	3DP Formulation		3DP Sample Nomenclature			
		Barrel 1	Barrel 2	Sample ID	Porosity	Flow Rate	Description
	(pbw)	(pbw)	(pbw)		(%)	(mL min^−1^)	
TMPe	72.9	46	26.9	FP_*H*_‐FR25	>80	0.25	High porosity
TDI	20.8	–	20.8	FP_*M*_‐FR50	75‐85	0.50	Medium porosity
DI water	1.6	1.6	–	FP_*L*_‐FR100	<75	1.00	Low porosity
DBTDL	1.9	1.9	–	AI‐3DP	–	–	AI‐designed
FSi	2.8	2.8	–				
**Total**	**100**	**52.3**	**47.7**				

Beyond material formulation and process control, this platform introduces AI as a design co‐pilot in foam design through a prompt‐based LLM architecture (Figure 1d). In this workflow, user queries or descriptive prompts (e.g., “tree‐like pore distribution” or “lotus leaf microchannels”) are translated into CAD geometries through iterative text‐to‐image‐to‐CAD generation and refinement loops. The multi‐agent LLM architecture, illustrated in Figures 1d_1_ and 1d_2_, integrates real SEM datasets and performance‐driven optimization criteria to generate designs that exhibit macroscale pore control alongside microscale stochasticity, enabling functionally graded porous architectures. This dual‐scale control is critical for balancing energy absorption, impact mitigation, and thermal management within a single material system. By automating what is traditionally a time‐intensive design–build–test cycle, the AI‐assisted framework enables near‐instant prototyping of complex bioinspired foams, significantly reducing development time from days or weeks to mere minutes. The resulting PU foams not only replicate natural motifs but also achieve superior thermo‐mechanical performance demonstrating low thermal conductivity (≈0.067 W m^−1^ K^−1^), high elastic recovery (>90% after 5000 loading cycles), and robust structural fidelity under compressive and cyclic loads. This synergy of AI‐driven architecture generation with static‐mixer‐enabled DIW printing establishes a versatile paradigm for rapidly developing adaptive porous materials targeted at next‐generation helmets, wearable thermotherapy systems, and lightweight thermal–mechanical insulation platforms.

### 2.2 Optimizing PU Synthesis with Static Mixing for Direct Ink Writing

The optimization of PU foam fabrication via static mixing‐enabled DIW addresses key limitations in processing rapidly curing thermoset systems. PU formation proceeds through a well‐established exothermic reaction between a polyol and a poly‐isocyanate in the presence of a catalyst, typically tin‐based DBTDL, and water when foaming is desired (Figure S1, Supporting Information), which has been widely reported elsewhere in the literature.^[^ ^42^, ^43^, ^44^ ^]^ In this study, we employ TMPe (a tri‐functional polyol with ethylene oxide units) and TDI (a mixture of 2,4‐ and 2,6‐isomers in an 80/20 ratio) as the isocyanate precursor to formulate a reactive PU‐ink tailored for DIW. A static mixing approach was implemented using a commercial laminar‐flow mixer (Figure S2, Supporting Information) to enable continuous homogenization of precursors just prior to deposition. This method overcomes the scalability and timing limitations associated with conventional batch‐based vortex mixing, where discontinuous operation and short pot life can lead to premature gelation and inconsistent form morphology during DIW.^[^ ^45^, ^46^ ^]^ In contrast to mechanical foaming techniques, which rely on batch stirring and face similar issues with uniformity and throughput, static mixers promote precise, continuous mixing via shearing, splitting, and recombining the fluid stream with a helical geometry, enabling programmable deposition on the processing platform.^[^ ^47^ ^]^ This continuous flow strategy aligns with the fast‐curing kinetics of PU systems, ensuring synchronized mixing and deposition that reduces void formation and enhances crosslinking consistency.^[^ ^48^ ^]^ Furthermore, unlike chemical foaming methods that depend on exact control over blowing agent decomposition timing, this in‐line reactive mixing approach inherently matches gas evolution with matrix solidification, yielding uniform cellular architectures well‐suited for scalable 3D printing.

**Figure 2:**
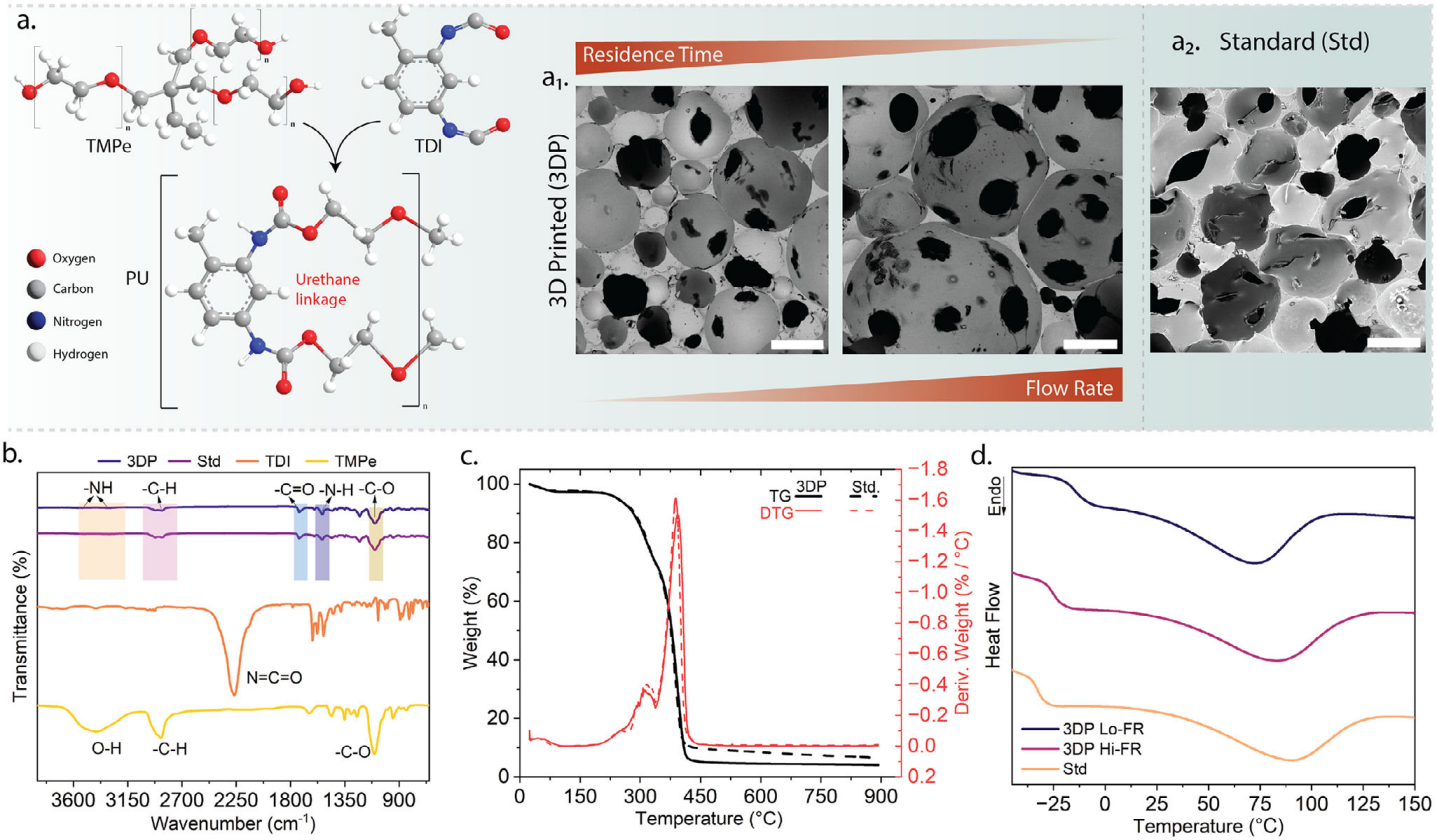
Characterization of PU foam formation and properties via DIW (3DP) and standard (Std) processing routes. a) A schematic representation of the exothermic reaction between TMPe and 2,4‐ and 2,6‐isomers TDI, forming urethane linkages during PU synthesis. SEM images illustrate the influences of residence time and flow rate on foam microstructure of (a_1_) 3DP compared to (a_2_) Std processing (scale bars ‐ 500 µm). The 3DP was ink writing with static mixing, while the Std samples were prepared via vortex batch mixing and casting. b) FTIR spectra of the precursor and formed foams confirm the disappearance of isocyanate bands and formation of urethane bonds in both 3DP and Std foams. c) Thermogravimetric analysis (TGA) compares the thermal stability of the 3DP and Std PU, showing comparable thermal degradation profiles and stability for both foam types, while d) differential scanning calorimetry (DSC) analysis highlights thermal transitions and segmental organization differences between Std and 3DP foams processed at low (Lo‐FR) and high (Hi‐FR) flow rates.

As illustrated in **Figure** **2**a, the hydroxyl groups (–OH) of TMPe, a polyether polyol, react with the isocyanate groups (–NCO) of TDI to form a PU matrix through the creation of urethane bonds (–NH–COO–). TMPe is typically trifunctional, containing three terminal –OH groups, though ethoxylation may introduce additional –OH sites or modify reactivity through steric and electronic effects. Here, –OH groups from TMPe function as reactive sites for urethane formation and promote extensive crosslinking to generate a 3D polymer network. The use of the tin‐based catalyst DBTDL accelerates the urethane‐forming reaction between hydroxyl and isocyanate groups, ensuring rapid gelation and enhancement of mechanical modulus. The water–isocyanate reaction, while less influenced by DBTDL, still proceeds under these conditions. Simultaneously, water present in the system reacts with excess–NCO groups to form carbamic acid intermediates that decompose into carbon dioxide (CO_2_) and amines; the CO_2_ acts as an in situ blowing agent, while the amines further react to form urea linkages, contributing to the foam's porous structure and mechanical strength.^[^ ^49^ ^]^ The ethoxylate chains on TMPe increase the molecular weight and flexibility of the polyol, which can slightly reduce the reactivity of the –OH groups compared to unmodified trimethylolpropane, thereby offering finer control over reaction kinetics and influencing the mechanical properties of the resulting PU foam through enhanced flexibility. Given that our DIW‐based manufacturing process requires precise control over reaction kinetics, a multi‐hydroxyl polyol such as the TMPe was selected to meet this requirement. It is well established that secondary hydroxyl groups react more slowly with isocyanate than primary hydroxyl groups, due to steric hindrance and reduced nucleophilicity. This difference in reactivity allows for greater control over PU network formation and enables precise tuning of foam properties through polyol selection.^[^ ^50^, ^51^ ^]^

To evaluate whether 3D printed foams match or exceed the structural robustness of conventionally prepared PU foams, we compared two distinct fabrication approaches. The standard (Std) foam was prepared via batch mixing and casting of TMPe, TDI, water, and DBTDL, following conventional protocols (see **Experimental Section**). In contrast, the 3D printed (3DP) foams were fabricated using a static mixer‐integrated DIW setup, enabling continuous homogenization and reactive extrusion. To assess structural differences, both foam types were analyzed using scanning electron microscopy (SEM), Fourier transform infrared spectroscopy (FTIR), thermogravimetric analysis (TGA), and differential scanning calorimetry (DSC). As shown in Figure 2a_1_, SEM images reveal the effect of residence time and flow rate on the pore architecture of the 3D printed foams. At lower flow rates (i.e., longer residence time), the printed foams exhibit more uniform, spherical pore structures with narrow size distributions, suggesting enhanced bubble stabilization during in situ foaming. In contrast, higher flow rates reduce residence time in the static mixer, leading to larger, more irregular pore morphologies, possibly due to incomplete homogenization and uneven gas nucleation. By comparison, the standard foam (Figure 2a_2_) exhibits a broader and less controlled pore size distribution, with signs of coalescence and anisotropy in cell shape. This variation likely results from uncontrolled kinetics in batch mixing, where uneven dispersion of blowing agents or delayed gelation can lead to inconsistent pore development.

Importantly, the predominantly open‐cell structure observed in 3DP foams arises from several synergistic processing factors inherent to the DIW‐static mixer system. The static mixer ensures thorough and continuous homogenization of reactive components, promoting uniform nuclei formation throughout the ink. During extrusion, the ambient pressure environment and relatively slower polymerization kinetics compared to closed‐system foaming allow gas pressures within the bubbles to equilibrate with the surroundings before the polymer network fully solidifies. This pressure normalization facilitates rupture of thin cell walls or windows between adjacent bubbles, yielding an interconnected, open‐cell morphology. Furthermore, the absence of elevated pressure and rapid cure that typically preserves closed cells enables cell walls to stretch and buckle without premature fracture, enhancing foam elasticity and resilience. The improved microstructural uniformity observed in the 3D printed samples supports the role of inline static mixing in promoting homogeneous polymerization and nucleation, which is critical for achieving reproducible foam architectures. These imaging findings highlight the advantage of residence‐time‐controlled 3D printing in tuning foam morphology, allowing for precise control over cell size and distribution. Thus, this 3DP control is essential for tailoring mechanical, thermal, or acoustic performance in application‐specific PU foams. Critically, the open‐cell architecture produced under these tailored conditions is highly relevant for applications requiring robust energy dissipation, as the interconnected cell walls can undergo repeated bending and buckling during deformation, a mechanism well known to underpin high hysteresis and elastic recovery in polymer foams.

As seen in Figure 2b, the FTIR spectra confirm the key chemical transformations involved during PU synthesis. The spectrum for TMPe features a strong, broad O‐H stretching band centered ≈3410 cm^−1^, characteristic of terminal hydroxyl groups, along with a distinct C–O stretching peak ≈1140 cm^−1^, indicative of its polyether backbone.^[^ ^52^, ^53^ ^]^ In contrast, the TDI spectrum exhibits a sharp absorption at 2270 cm^−1^, corresponding to the isocyanate (─N═C═O) functional group, with negligible O–H or N─H signals, confirming the presence of unreacted isocyanate.^[^ ^54^, ^55^ ^]^ Upon reaction, both the Std and 3DP PU foams exhibit the disappearance of the isocyanate peak at 2270 cm^−1^, indicating full consumption of TDI. New absorptions appear in the 3300–3400 cm^−1^ region, attributed to N‐H stretching of the urethane linkages, and a strong C═O stretch emerges near 1720 cm^−1^, characteristic of the carbamate (urethane) group.^[^ ^53^ ^]^ The retention of the C–O stretch ≈1140 cm^−1^ in retained in both spectra, reflecting the continued presence of the polyether segment from TMPe. In addition, a band near 1540 cm^−1^, associated with N–H bending and C–N stretching (amide II region), further confirms urethane bond formation.^[^ ^56^ ^]^ The high similarity between the Std and 3DP spectra indicates that both fabrication methods yield chemically equivalent PU networks with little detectable residual monomers. These findings further demonstrate that the static mixer‐assisted DIW process effectively facilitates complete and homogeneous PU formation, comparable to conventional batch processing.

The thermal decomposition behavior of the 3DP and Std‐processed PU foams was studied using TGA. As seen in Figure 2c, a small weight loss is observed at temperatures between 100 and 200 °C, attributed to the evaporation of residual moisture, solvents, and even low‐molecular‐weight volatiles. This is followed by a significant weight loss between 300 °C and 400 °C, marked as the first degradation step, corresponding to the thermal breakdown of urethane linkages and releases carbon dioxide, amines, and hydrocarbon fragments.^[^ ^57^, ^58^ ^]^ This is characterized by a sharp decrease in the weight loss curve and a maximum degradation rate at ≈380 °C, as reflected in the derivative weight curve (red curve). A secondary degradation step occurs beyond 400 °C, associated with the degradation of polyether polyol chains and further breakdown of char‐like residues ^[^ ^59^ ^]^. Notably, both the 3DP and Std samples exhibit highly similar degradation profiles, indicating that the reactive extrusion process used in the DIW does not adversely affect the thermal stability of the resulting PU foams. This also suggests that the chemical integrity and crosslinking density of the 3D printed PU foam are well preserved, even under elevated thermal conditions.

The DSC thermograms in Figure 2d compare the thermal transition of PU foams fabricated using different processing parameters/methods. All samples exhibit a sub‐ambient baseline shift between –25 and 0°C, which corresponds to the glass transition of the soft segment domains, confirming the presence of phase‐separated morphology across all formulations. For the 3DP Lo‐FR (low flow rate <0.5 mL min^−1^) sample, a broad thermal transition centered near 80°C is observed, which likely corresponds to a relaxation or disordering of the hard segment domains. The broadness of this feature suggests a greater degree of micro‐phase mixing or heterogeneity, potentially introduced by the extended residence time in the static mixer. In contrast, the 3DP Hi‐FR (high flow rate >0.5 mL min^−1^) sample exhibits a sharper and slightly earlier transition in the same temperature region. This behavior is indicative of a more defined and phase‐separated hard segment structure, likely a result of reduced mixing time and more rapid deposition, which limits chain linkage and interaction during in situ polymerization. The Std sample displays the narrowest and most defined transition in the hard segment region, suggesting a more uniform microphase‐separated morphology. This is consistent with the efficient dispersion and controlled curing typically achieved through batch mixing. Overall, the flow rate and residence time in static mixing have shown a measurable impact on segmental organization and thermal behavior in 3DP PU foams, without disrupting the essential phase‐separated nature of the material.

### 2.3 Rheology of Preformed PU mixture for DIW

**Figure 3:**
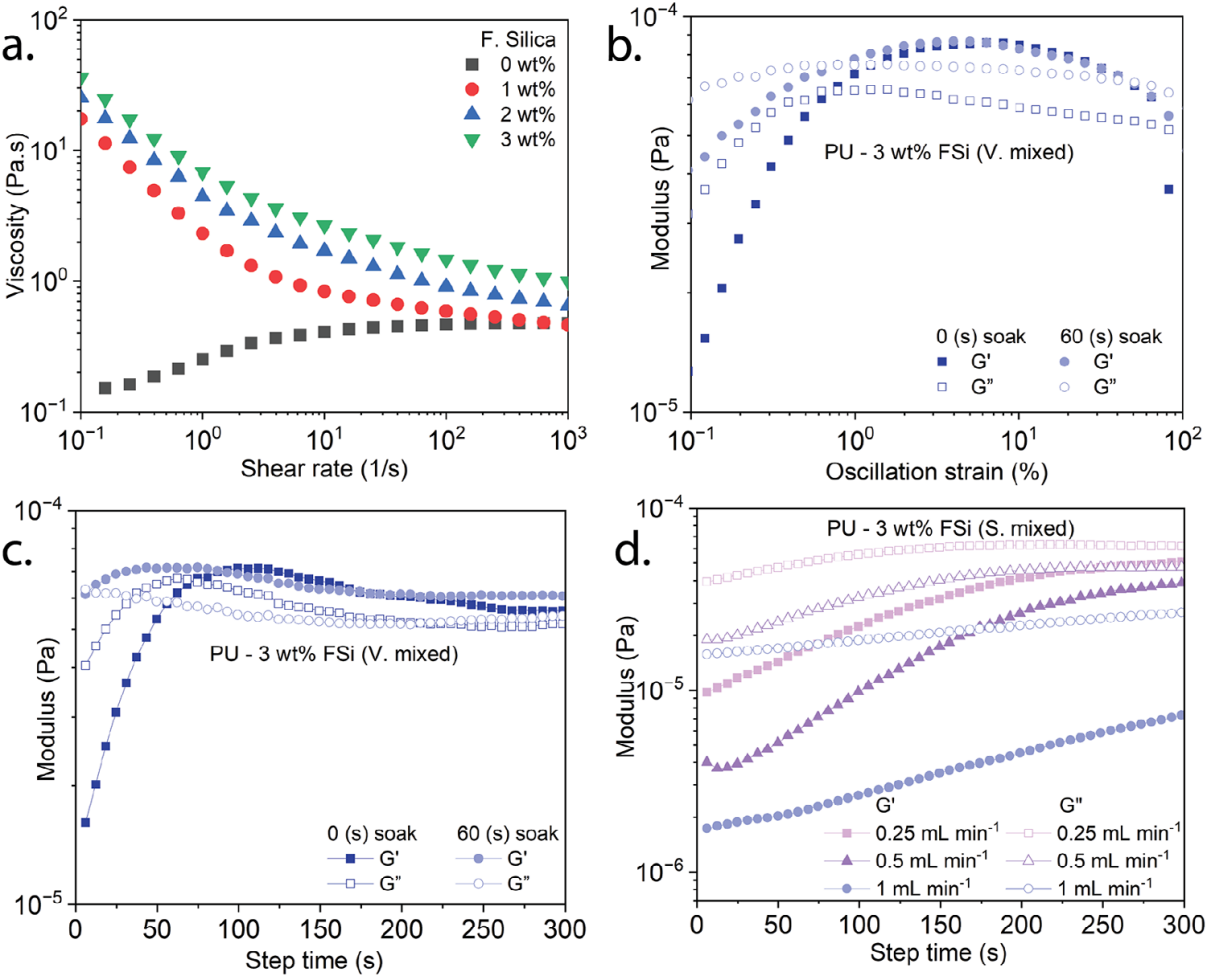
Rheological characterization of preformed PU mixtures with varying FSi loading and mixing methods. a) Shear viscosity profiles of vortex‐mixed PU formulations containing 0, 1, 2, and 3 wt% FSi, showing shear‐thinning behavior with increased viscosity at higher filler content, which improves printability for DIW. b) Oscillatory strain sweep of vortex‐mixed PU with 3 wt% FSi, evaluated at 0 s and 60 s soak times, highlighting the effect of pre‐shear aging on viscoelastic moduli (G, G) as a function of strain amplitude. c) Time‐dependent evolution of G and G during small‐amplitude oscillatory shear for vortex‐mixed PU (3 wt% FSi), comparing the kinetics of gelation with and without a 60 s pre‐shear soak. d) Viscoelastic response of statically mixed PU (3 wt% FSi) at different flow rates (0.25, 0.5, and 1.0 mL min^−1^, demonstrating the influence of flow rate on gelation behavior and storage modulus development over a 300 s time window.

Achieving a well‐defined shear‐thinning response is essential for successful DIW of PU foams. As shown in **Figure** **3**a, the preformed PU mixture without FSi (0 wt% FSi) exhibits near‐Newtonian behavior with consistently low viscosity across shear rates, rendering it unsuitable for extrusion‐based printing. To address this limitation, FSi was introduced as a thixotropic agent to tailor the ink's rheological profile, promoting shear‐thinning behavior critical for controlled deposition.^[^ ^60^, ^61^ ^]^ FSi particles form a transient 3D network through weak interparticle interactions, which breaks down under shear stress, thereby reducing viscosity during flow and recovering structure at rest. The onset of pronounced shear‐thinning behavior was observed at 1 wt% FSi and further enhanced with increasing loading up to 3 wt%, enabling smooth and stable extrusion through a nozzle under practical flow conditions. This trend confirms that the incorporation of FSi is not only beneficial but essential for inducing desirable viscoelastic properties suitable for DIW. Based on these observations, the 3 wt% FSi formulation was selected as the optimized ink for 3D printing and subsequent analysis.

In extrusion‐based 3D printing, such as DIW, achieving shear‐thinning behavior is essential for ensuring smooth ink flow through the nozzle, while maintaining sufficient post‐extrusion mechanical stability is equally important to preserve the printed shape during layer‐by‐layer construction. This structural stability is governed by the ink's viscoelastic properties, particularly the storage modulus (G), which reflects the material's ability to maintain shape and resist deformation after extrusion. As shown in Figure 3b, oscillatory strain sweeps of vortex‐mixed PU formulations with 3 wt% FSi demonstrate that a 60‐s soak time enhances the solid‐like character (evidenced by higher G values) compared to samples without a soak, shifting the viscoelastic response closer to the gel state. This indicates that short pre‐conditioning periods promote microstructural reorganization and early‐stage crosslinking. The time‐dependent evolution of viscoelasticity in Figure 3c further supports this observation, both G and G increase sharply with time under constant small‐amplitude oscillatory strain, reflecting progressive gelation as isocyanate (NCO) and hydroxyl (OH) groups react. The point where G surpasses G observed between 100 and 150 s, marks the liquid‐to‐solid transition, which is critical for ensuring each printed filament can support subsequent layers without collapsing. This dynamic evolution of viscoelastic properties emphasizes the importance of tuning residence time and pre‐shear conditions to synchronize polymerization and foaming kinetics during DIW, directly informing the optimized flow‐rate conditions examined in Figure 3d.

For DIW of PU foams, achieving the appropriate balance between mixing, gelation, and foaming is essential for producing inks that are both extrudable and structurally stable upon deposition. As schematically depicted in (Figure 1), our setup leverages a static mixer (, Supporting Information) to continuously and homogeneously blend reactive components just prior to extrusion, thereby synchronizing chemical crosslinking and gas bubble nucleation. The flow rate plays a crucial role in this process. As shown in Figure 3d, lower flow rates (e.g., 0.25 mL min^−1^) result in a more rapid increase in G over time, indicating accelerated network formation and solidification, as shown in Figure 3d. This enhancement is attributed to prolonged residence time in the static mixer, which facilitates more complete mixing and increased pre‐polymerization prior to nozzle exit. Conversely, higher flow rates reduce mixing time and delay modulus development (Figure S3 Supporting Information). As a result, the synchronized mixing, polymerization, and foaming dynamics must be carefully tuned to enable DIW of PU foams with optimized rheological properties and structural fidelity.

### 2.4 Bioinspired, AI‐Facilitated Foam Architecture 3D Printing

**Figure 4:**
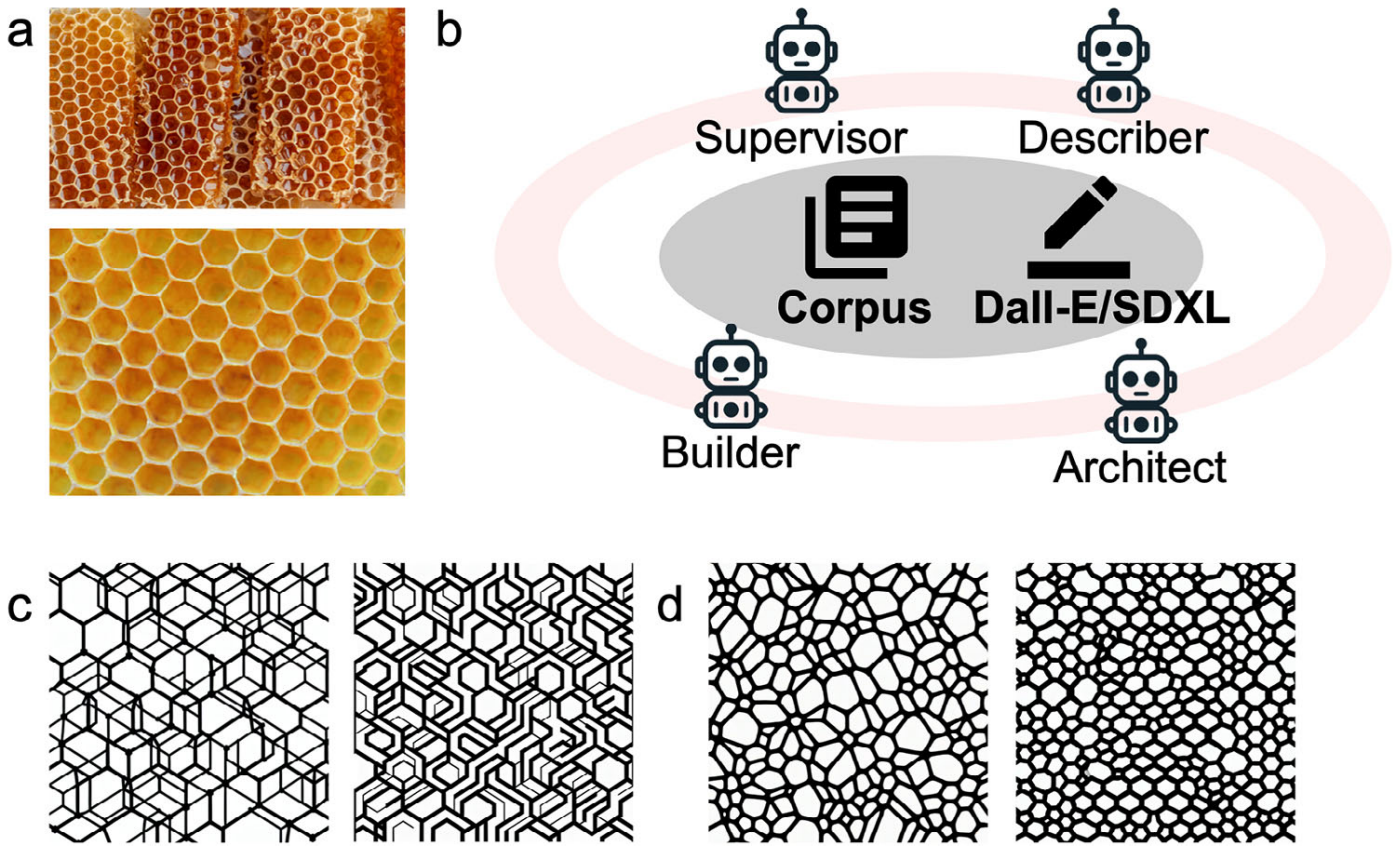
The design pipeline for AI‐generated printable models. a) The input consists of nature‐inspired honeycomb images, featuring both tubular and hierarchical architectures. b) The multi‐agent generative framework consists of four agents, namely, Supervisor, Describer, Architect, and Builder, working collaboratively. The Builder operates using either DALL‐E or SDXL, and all agents share access to a common corpus. c) Designs generated by DALL‐E show fine‐scale, repetitive features with dense line networks. d) Designs generated by a fine‐tuned SDXL model exhibit cleaner geometries and are directly suitable for 3D printing.

Conventional foam design strategies primarily target microscale porosity, which restricts their ability to exploit the structural complexity and multiscale integration necessary for advanced additive manufacturing. In contrast, we present an AI‐driven design paradigm leveraging a custom‐built multi‐agent framework that integrates GPT‐4o and a fine‐tuned Stable Diffusion XL (SDXL) model to rapidly generate hierarchical, print‐ready structures suitable for DIW fabrication (**Figure** **4**).^[^ ^62^ ^]^ As illustrated in Figure 4b, the framework operates through four AI agents—Describer, Architect, Builder, and Supervisor—collaborating via prompt chaining and shared memory access. The Describer interprets morphological features from bioinspired inputs (e.g., honeycomb motifs in Figure 4a), while the Architect translates these descriptors into generative prompts. The Builder, powered by either DALL‐E or a fine‐tuned SDXL model, creates structural candidates, and the Supervisor evaluates and refines outputs to ensure geometric fidelity and print compatibility. The fine‐tuned SDXL model is particularly trained to generate structurally coherent, print‐compatible designs, addressing limitations in geometric fidelity seen in default DALL‐E outputs (Figure 4c,d). This AI‐assisted approach accelerates the traditionally iterative design–fabrication cycle, enabling rapid exploration of bioinspired motifs and expanding the design space for multiscale, application‐specific foams.^[^ ^62^ ^]^

**Figure 5:**
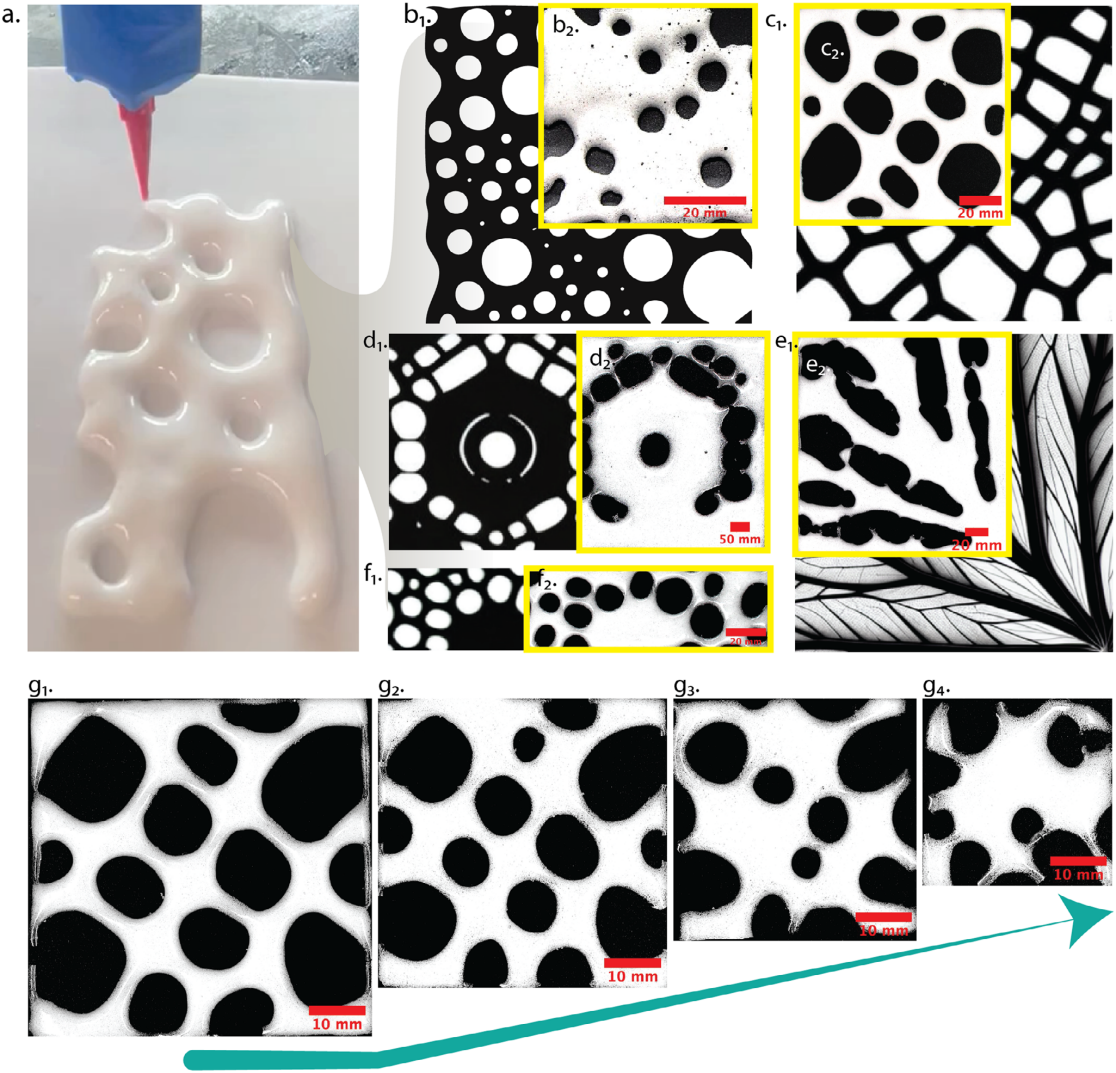
a) DIW of the preformed PU mixture during deposition. (b_1_, c_1_, d_1_, e_1_, f_1_) AI‐generated 2D architectural motifs derived from natural inspirations, including vascular and venation‐like patterns, produced using the multi‐agent LLM–SDXL framework. (b_2_, c_2_, d_2_, e_2_, f_2_) Corresponding optical images of the printed PU foams validate accurate translation of digital designs into physical, hierarchical porous structures with preserved pore connectivity and spatial gradients. (g_1_–g_4_) Resolution scaling study illustrating the DIW system's fidelity at progressively smaller feature sizes; finer pores (g_3_, g_4_) exhibit partial loss of definition due to bubble coalescence and expansion dynamics during reactive foaming.

**Figure** **5** exemplifies the integration of AI‐driven design and DIW‐based manufacturing to create hierarchical PU foams inspired by natural architectures. Leveraging motifs such as lotus leaves, vascular venation, and leaf microchannels, the multi‐agent LLM–SDXL framework generates bioinspired pore geometries that combine multiscale branching with interconnected networks ideally suited for multifunctional performance. These digital blueprints (Figure 5b_1_‐f_1_) are seamlessly translated into physical foams (Figure 5b_2_‐f_2_), faithfully preserving intended pore connectivity and spatial gradients across multiple length scales‐ nanopores (1–1000 nm), micropores (1–62.5 µm) mesopores (62.5 µm–4 mm), and macropores (>4 mm), (not following standardized IUPAC conventions, but is followed in some engineering/scaffolds/ceramics context). This hierarchical structuring is critical for multifunctional performance, as larger macropores localize deformation and absorb mechanical energy for improved impact attenuation, while smaller pores increase interfacial area, disrupting heat flow paths and enabling enhanced surface functionality.^[^ ^8^, ^63^, ^64^ ^]^ The ability to rapidly manipulate pore hierarchy and distribution with digital precision represents a significant advance over conventional foams, showcasing how the creative collaboration between digital intelligence and materials science can deliver tailored, high‐performance porous architectures.

To further evaluate the fidelity of the DIW process across multiple length scales, a resolution study (Figure 5g_1–4_) was performed. When combined with AI‐generated architectures, the DIW platform reliably preserves structural integrity at macroscale features; however, resolution limits emerge as pore dimensions approach the lower mesopore or micropore range. At these finer scales, capillary‐driven bubble coalescence, wall thinning, and anisotropic expansion dominate, leading to partial feature merging and loss of fidelity, as observed in Figure 5g_3–4_.^[^ ^65^ ^]^ These challenges arise from the intrinsic coupling between ink rheology, curing kinetics, and gas evolution dynamics inherent to the reactive foaming process. Additionally, factors not directly captured in the figure, such as localized thermal gradients across the build platform, transient backpressure in the nozzle due to variable feature density, and minor fluctuations in ambient humidity, can all influence deposition stability and final morphology. Rapid 3D printing deposition at small scales may also outpace crosslinking, resulting in insufficient gel strength to retain complex features. Conversely, slower print speeds are needed for fidelity risk, premature curing, and nozzle clogging, highlighting a delicate balance between kinetics and flow. Therefore, while macroscale DIW structuring is robust and reproducible, extending control into the nanoscale regime requires further innovation in real‐time rheological tuning, adaptive extrusion algorithms, and potentially external stimuli (e.g., UV curing or electric fields) to stabilize fine features. These insights underline the critical need to co‐optimize formulation chemistry, machine parameters, and environmental control for hierarchical foam manufacturing at ultra‐fine resolution.^[^ ^66^, ^67^ ^]^

### 2.5 Mechanical Properties of DIW PU Foams

**Figure 6:**
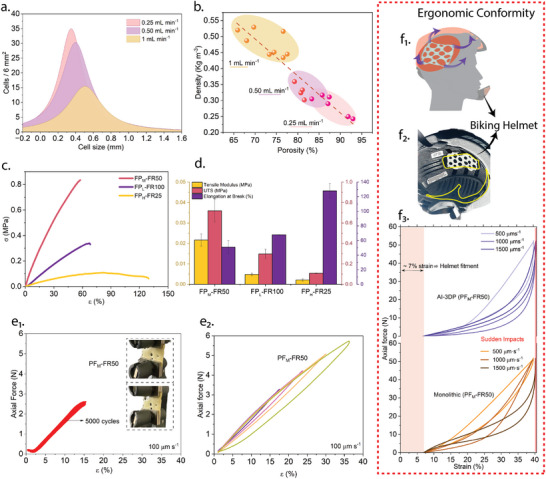
a) Cell size distributions measured over a 6 mm^2^ area across different DIW flow rates, demonstrating the influence of flow rate on cellular morphology. b) Relationship between porosity and density, showing that higher flow rates yield lower porosity and denser foam structures. c,d) Mechanical performance of printed foams as a function of porosity: FP_*M*_‐FR50 exhibited the highest tensile modulus and ultimate tensile strength (UTS), attributed to its optimized balance between porosity and pore size. In contrast, FP_*H*_‐FR25, with the highest porosity (higher than 80%), showed the greatest elongation at break, indicating superior elasticity. Mechanical properties of FP_*M*_‐FR50, with the (e_1_) cyclic tensile tests of AI‐3DP foams at 100 µm s^−1^ over 5000 cycles confirm mechanical durability with stable stress‐strain response; and (e_2_) tensile behavior at various displacement rates confirms the elastic performance under varying loading conditions. (f_1_) Application of AI‐3DP foam structures for impact mitigation in protective headgear, with the schematic showing the ergonomic conformity of AI‐3DP foam inserts to human cranial anatomy, enabling zonal energy absorption. (f_2_) Image of a commercial biking helmet retrofitted with PU foam inserts (highlighted in yellow) at impact‐prone zones to enhance protection, particularly in regions aligned with MIPS technology. (f_3_) Axial force vs. strain curves comparing the performance of AI‐3DP foams and bulk PU foams at different strain rates (500, 1000, and 1500 µm s^−1^). The AI‐3DP foams exhibit higher stiffness and impact energy dissipation above helmet‐fit strain thresholds ( 7%), making them ideal for sudden impact scenarios such as cycling accidents.

The cellular morphology, porosity, and mechanical behavior of 3D‐printed PU foams are strongly influenced by the applied flow rate during DIW. **Figure** **6**a illustrates the relationship between pore size distribution and pore density for three flow rates (0.25, 0.5, and 1 mL min^−1^), evaluated over a standardized 6 mm^2^ area and averaged across triplicate samples. The pore size distributions across all flow rates are unimodal, but a prominent shift is observed as the flow rate increases. At the lowest flow rate (0.25 mL min^−1^), foams exhibit a high density of small pores centered around ≈0.4 mm. This suggests enhanced nucleation due to extended residence time within the static mixer, promoting homogeneous mixing and inhibiting premature coalescence. This refinement arises from prolonged residence time in the static mixer, which promotes homogeneous precursor mixing and partial pre‐polymer crosslinking before gas expansion. Enhanced nucleation and rapid gelation under these conditions suppress Ostwald ripening, a thermodynamically driven coarsening process where larger bubbles grow at the expense of smaller ones to minimize interfacial energy.^[^ ^68^ ^]^ In contrast, at higher flow rates (0.5 and 1 mL min^−1^), shortened residence time limits precursor homogenization, delaying gelation and allowing bubbles to coalesce and grow, thereby reducing pore density and increasing average cell size. For clarity, the present work employs specific labels to denote flow rates in these foams: FP_*H*_‐FR25 indicates the lowest flow rate of 0.25 mL min^−1^, FP_*M*_‐FR50 corresponds to an intermediate flow rate of 0.50 mL min^−1^, and FP_*L*_‐FR100 represents the highest flow rate of 1.00 mL min^−1^.

SEM imaging (Figure S4, Supporting Information) confirms that all formulations retain an open‐cell architecture regardless of flow rate, with interconnected windows formed through rupture of thin walls between adjacent bubbles as internal pressure equilibrates with ambient conditions. This morphology is characteristic of ambient‐pressure foaming in DIW, where slow curing enables cell wall thinning and coalescence into continuous porous networks. The degree of structural uniformity, however, strongly depends on viscoelastic evolution during processing. Rheological data (Figure 3d) show that low flow rates (0.25 mL min^−1^) accelerate the storage modulus (*G*′) crossover within 100–150 s, signifying the transition to a solid‐like state necessary for shape retention and fine pore stabilization. In contrast, higher flow rates (0.5–1 mL min^−1^) reduce residence time in the static mixer, delaying crosslinking and maintaining a viscous‐dominated response (elevated loss modulus, *G*″, with no *G*′ crossover within 300 s). This delayed gelation allows bubbles to grow and merge, leading to coarser pores and reduced cell density. The observed pore size shift from densely nucleated ≈0.4 mm pores at low flow rates to larger, more polydisperse structures at 1 mL min^−1^, is consistent with Ostwald ripening behavior, wherein larger bubbles grow at the expense of smaller ones to minimize interfacial energy.^[^ ^68^, ^69^ ^]^ Thus, these findings demonstrate that residence‐time control via flow rate serves as a critical design handle for tuning pore architecture, enabling the fabrication of foams ranging from finely nucleated, high‐porosity networks to coarser, mechanically distinct structures suited for varied functional requirements.

These morphological changes (Figure S4, Supporting Information) translate directly into variation in porosity and bulk density of the printed foams, as shown in Figure 6b. A clear inverse relationship is observed: as flow rate increases, porosity decreases while density increases. Specifically, foams printed at 0.25 mL min^−1^ exhibit porosities above 85% and higher densities lower than 0.3 kg m^−3^, whereas those at 1 mL min^−1^ exhibit porosities averaging 70% with corresponding densities higher than 0.45 kg m^−3^. This behavior highlights the critical role of flow‐mediated residence time and curing synchronization. At slower flow rates, extended residence within the static mixer promotes thorough precursor homogenization and earlier crosslinking, locking in smaller, densely packed pores before significant gas expansion. Conversely, higher flow rates shorten residence time, delaying gelation and enabling prolonged bubble coalescence and expansion, producing larger pores and less uniform networks. These findings establish flow rate as a deterministic design parameter for tuning hierarchical foam morphology and bulk properties, offering a direct pathway to tailor mechanical cushioning and thermal insulation performance for specific applications.

Figure 6c,d presents the mechanical response of 3D‐printed PU foams as a function of processing‐induced porosity and flow‐rate‐controlled cellular morphology. Here, the open‐cell morphology inherent to these foams ensures that macroscopic deformation is dominated by bending and stretching of interconnected struts rather than fracture of closed‐cell walls, enabling high strain tolerance and energy dissipation. The uniaxial tensile stress–strain curves in Figure 6c reveal distinct behaviors across the three formulations, FP_*M*_‐FR50, FP_*L*_‐FR100, and FP_*H*_‐FR25, demonstrating how microstructure differences influence stiffness, strength, and extensibility. The FP_*H*_‐FR25 sample, produced at the lowest flow rate (0.25 mL min^−1^), features a highly porous architecture, resulting in the highest elongation at break (140%) but the lowest tensile modulus and ultimate tensile strength (UTS). This soft, compliant behavior is attributed to sparse, interconnected pores that enable large deformations without structural collapse. Conversely, FP_*L*_‐FR100, printed at the highest flow rate (1 mL min^−1^), demonstrates enhanced stiffness and UTS due to a denser foam structure with reduced pore expansion; however, it suffers from reduced stretchability, indicative of a more brittle failure mechanism. Between these extremes, the FP_*M*_‐FR50 formulation, produced at an intermediate flow rate (0.5 mL min^−1^), exhibits a balanced mechanical profile that combines moderate stiffness with considerable ductility. Notably, the monotonic trends observed in pore size distribution and porosity across Figure 6a,b are directly paralleled by the gradation in mechanical properties in Figures 6c, collectively reinforcing a predictable structure– property paradigm. This alignment underlies the rationale for selecting FP*M*‐FR50 as an optimized candidate for further demonstration, effectively balancing stiffness, strength, and extensibility to meet the demands of wearable protection systems. As shown in Figure 6d, the bar graph quantifies this trade‐off by displaying tensile modulus, UTS, and elongation at break for each formulation. The FP_*M*_‐FR50 foam emerges as an optimized candidate for applications requiring a combination of energy absorption, deformability, and structural resilience, making it especially relevant for wearable protection systems.

To assess the mechanical resilience and energy management of the optimized FP_*M*_‐FR50 formulation, cyclic tensile testing was performed under standardized laboratory conditions (Figures 6e_1_,e_2_). Under 5000 loading cycles at a fixed strain amplitude of 14%, a loading rate of 100 µm s^−1^, and ambient temperature of 19–20°C, the stress–strain response exhibited narrow, highly repeatable hysteresis loops, indicative of minimal energy loss and excellent elastic recovery exceeding 90% over extended cycling. Quantitative analysis indicated that the hysteresis energy dissipation at this strain level was ≈67 J m^−3^ per cycle, with negligible residual strain accumulation. This result confirms that the foam structure effectively resists permanent deformation under moderate cyclic loading and retains its mechanical integrity over repeated use. As the maximum strain amplitude was incrementally increased to 35%, substantial increases in both the hysteresis loop area and peak tensile force were recorded, with dissipated energy rising from 67 to nearly 967 J m^−3^. This indicates the transition to enhanced energy‐absorbing mechanisms, notably pore wall buckling and local plastic deformation, contributing to stress distribution and efficient energy absorption under higher strains. There is clear evidence that the hierarchical architecture, macropores bounded by microporous walls, directly enables stable load distribution and suppression of catastrophic structural collapse, as observed by the absence of abrupt transitions or failure through the entire operational window. These findings, consistent with recent mechanics‐of‐lattice studies, demonstrate that the 3D‐printed FP_*M*_‐FR50 foam, with its strain‐adaptive energy dissipation and robust durability, is highly suitable for advanced PPE applications requiring dependable mechanical performance under cyclic and impact loads.^[^ ^70^, ^71^ ^]^

The practical integration of AI‐architected, 3D‐printed foams into protective equipment is demonstrated in Figure 6f_1–3_, with a specific focus on their application in cycling helmets. The schematic illustration (Figure 6f_2_) emphasizes the ergonomic conformity achieved through custom‐designed foam structures that align with human cranial anatomy. This anatomical matching ensures uniform contact and optimized pressure distribution across the head, thereby enhancing comfort and reducing fatigue during prolonged use. The photograph in Figure 6f_2_ shows the physical integration of such architectured foam into a commercial helmet, where the insert has been strategically placed in regions aligned with MIPS (Multi‐directional Impact Protection System) components. This design aims to absorb linear impact and permit controlled relative movement, thereby mitigating rotational forces experienced during oblique impacts.^[^ ^72^ ^]^ Traditionally, helmet systems rely on hard outer shells combined with expanded polystyrene (EPS) or expanded polypropylene (EPP) liners to absorb high‐energy impacts. Premium models incorporate MIPS technology to reduce rotational acceleration. However, these conventional systems often lack tunability in low‐velocity impact regimes or ergonomic compliance. By precisely tuning the flow rate during DIW, our AI‐optimized foam architectures can be fabricated with distinct mechanical profiles, enabling customized strain responses under different loading conditions.

This tunability is supported by compressive testing results (Figure S6, Supporting Information), which indicate that flow rate directly modulates foam compressive modulus and energy absorption capacity due to changes in pore size and wall thickness. This provides dual advantages: increased comfort during routine use and enhanced energy dissipation under sudden impacts. Such foam designs not only complement but may also enhance existing MIPS systems by introducing a micro‐architected energy‐absorbing interface that mimics cranial motion and supports rotational flexibility.^[^ ^8^, ^73^ ^]^ Quantitative comparison of impact response is presented in Figure 6f_3_, where axial force versus strain curves compare AI‐3DP foams and bulk PU foams (both based on the FP_*M*_‐FR50 formulation) across strain rates of 500, 1000, and 1500 µm s^−1^. The operational helmet‐fitment strain range (≈7%) is highlighted in pink, where both foams show similar compliance, ensuring wearer comfort during routine use. Beyond this threshold, mimicking sudden impact conditions, the AI‐3DP foams display markedly higher stiffness and energy dissipation, attributed to multiscale deformation mechanisms including pore collapse and wall buckling. These mechanical enhancements are a result of the hierarchical structuring achieved through AI‐guided design and validated by compressive testing (Figure S6, Supporting Information), collectively demonstrating how tailored pore morphology and architectural connectivity influence strain‐stiffening and energy absorption in the foams. The integration of hierarchical micro‐architecture enables the foam to provide appropriate compliance at low strains for comfort and efficient energy dissipation under higher strains relevant to impact. This systematic approach, linking compressive performance with controlled hierarchical design, highlights the suitability of AI‐developed foams for advanced helmet liners, where both mechanical safety and ergonomic fit are essential.^[^ ^8^, ^73^ ^]^

### 2.6 Thermal Properties of DIW PU Foams

**Figure 7:**
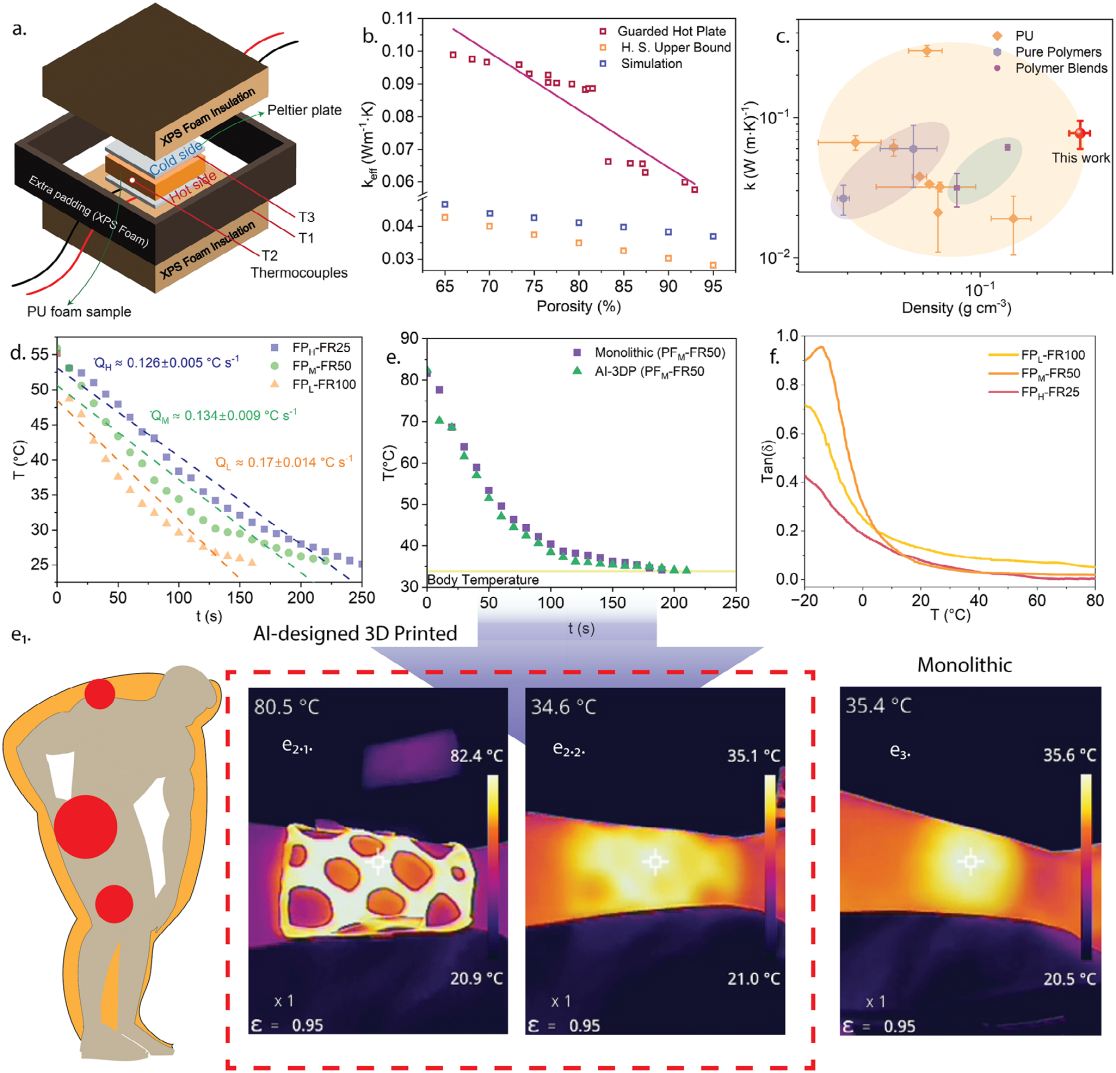
a) Schematic of the guarded hot plate setup used to evaluate thermal conductivity (TC) of printed PU foam samples. b) Measured TC values plotted against porosity, showing a linear decrease with increasing porosity (between 65% and 95%), consistent with Hashin–Shtrikman (H.S.) upper bound theory and simulations. c) Ashby‐style plot comparing thermal conductivity (k) of 3DP PU foam in this work with literature‐reported values for PU foams, pure polymer, and polymer blends.^[^ ^67^, ^77^, ^78^, ^79^, ^80^, ^81^, ^82^, ^83^, ^84^, ^85^, ^86^, ^87^, ^88^ ^]^. d) Quantified heat loss behavior across PU architectures showing the influence of the influences of porosity on thermal dissipation rates. e) Temperature–time profiles comparing bulk PU foam versus AI‐architected (AI‐3DP) PU foams, demonstrating comparable heat retention after thermal conditioning at 100 °C. (e_1–3_) Infrared thermographs from a qualitative demonstration of thermal transfer from PU foams to a human subject, comparing AI‐3DP versus bulk PU foams. The AI‐3DP structure exhibits superior thermal insulation, indicated by lower temperature propagation on the subject's body. f) Tan δ profiles from dynamic mechanical analysis (DMA) under 100 °C thermal loading, illustrating distinct viscoelastic responses for each foam type.

**Figure** **7** depicts the thermal properties of the printed PU foam structures through thermal conductivity measurements, cooling curve analysis, and thermotherapy to explain the structure–property relationship. Figure 7a shows the custom‐built guarded hot plate apparatus used to quantify the effective thermal conductivity (*k*_eff_) of foams made at varying flow rates (Figure S5, Supporting Information).^[^ ^74^ ^]^ Figure 7b demonstrates how (*k*_eff_) systematically decreases as porosity increases from from 65% to 95%, with values dropping from ≈0.105–0.10 to as low as 0.025–0.067 W m^−1^ K^−1^, highlighting the potential for enhanced thermal insulation in higher porosity foams. This trend is primarily attributed to the growing fraction of thermally insulating air pockets as porosity increases. The measured *k*_eff_ values are consistently above both the Hashin–Shtrikman (H.S.) upper bound predictions and simulations (Figures S6 and S7, Supporting Information),^[^ ^75^, ^76^ ^]^ likely due to real‐foam features such as open‐cell connectivity, irregular pores, and possible cell wall thinning, which are not fully captured in idealized models. This behavior offers clear design flexibility in tuning insulation performance through control of porosity during printing. Figure 7c presents a comparison of thermal conductivity (*k*) versus density for the 3D‐printed PU foams, alongside values reported for conventional PU foams, pure polymers, and polymer blends. The 3DP PU foam samples in this work cluster at lower density and lower thermal conductivity compared to most conventional PU systems, occupying a segment of the property space characterized by lightweight construction and efficient thermal insulation among polymer‐based materials. However, their *k* and density values remain within the broader range of established PU foams and do not reach the ultralow conductivities reported for advanced materials such as aerogels or nanocellular polymers. Rather than surpassing the state‐of‐the‐art, the results indicate that the 3DP process enables practical access to lightweight, low‐density foams with thermal conductivities suitable for insulation applications. This ability to position material properties in a desired region of the *k*–density landscape demonstrates the usefulness of controlled, architectured foaming for customizable insulation performance, within the context of established polymer foam materials.

The cooling behavior of the printed PU foams was systematically examined through transient thermal dissipation analysis, as illustrated in Figures 7d,e. These plots reveal the critical influence of flow rate on heat retention and thermal dissipation characteristics. Specifically, Figure 7d quantifies the cooling rate *Q*_*H*_ as a function of time using infrared thermography, showing a clear dependence on flow rate‐controlled pore architecture. The FP_*H*_‐FR25 sample (fabricated at the lowest flow rate of 0.25 mL min^−1^) demonstrated the slowest cooling rate at *Q*_*H*_ = 0.126 ± 0.005 °C s^−1^, indicating superior thermal retention corresponding to its highest porosity (≈85%) and finest pore size distribution. The FP_*M*_‐FR50 sample (0.5 mL min^−1^) displayed an intermediate heat loss rate of 0.134 ± 0.009 °C s^−1^ with a porosity near 78%,, while the FP_*L*_‐FR100 sample (1.0 mL min^−1^) exhibited the fastest heat dissipation at 0.17 ± 0.014 °C s^−1^, reflecting diminished insulation performance with the lowest porosity (≈70%). These results highlight a tradeoff governed by foam architecture: although higher porosity generally correlates with lower thermal conductivity, the accompanying increase in average pore size at high flow rates can offset insulation benefits. As previously discussed in Figure 3d, increased flow rate reduces the gelation time and structural integrity, potentially inducing polymer drainage from the pore walls and triggering wall collapse. This coalescence leads to enlarged pore domains, which serve as thermal bridges that accelerate heat loss. Consequently, even though the FP_*L*_‐FR100 foam exhibits high porosity, its larger pore sizes undermine its insulating capabilities. By contrast, the FP_*H*_‐FR25 maintains a denser, finer cellular morphology, resulting in enhanced thermal insulation due to reduced convective and conductive heat transfer pathways.

To further probe application‐relevant performance, comparative thermotherapy assessments were conducted between bulk PU foam and AI‐3DP foams fabricated with identical PF_*M*_‐FR50 parameters, selected for their optimal balance between mechanical integrity and thermal retention. As shown in Figure 7e, both samples were preconditioned at 100 °C, after which their surface temperatures reached ≈85 °C. The subsequent cooling curves displayed nearly identical decay trajectories, demonstrating that the AI‐3DP foams maintain thermal energy storage comparable to their bulk PU foam counterparts despite possessing greater specific surface area and architectural complexity. This preserved insulation performance is attributed to the engineered multiscale pore hierarchy, which suppresses thermal losses by regulating conductive and convective pathways within the foam matrix. Thermal imaging data (Figure 7e_2.1–2.2_) further highlights spatial heat distribution: immediately after heating, the AI‐3DP foam exhibited localized hot zones up to 82.4 °C contrasted with cooler regions near 20.9 °C, demonstrating its capability for thermal channeling. After cooling, this structured thermal profile persisted with a range of 34.6–35.1 °C, whereas the bulk PU foam sample showed a uniform but less controllable thermal distribution (35.4–35.6 °C). Such directed heat management is particularly valuable for therapeutic applications, as illustrated by the human‐subject demonstration in Figure 7e_2.1–2.2_, where localized heat delivery is essential for treating specific muscle groups and joint conditions.^[^ ^89^, ^90^ ^]^ As can be seen from the above discussions, Figure 7d,e underscores the importance of balancing porosity, pore morphology, and architectural control to achieve high‐performance thermally insulating foams with application‐specific heat retention characteristics.

The dynamic mechanical analysis (DMA) of the 3D‐printed PU foams, shown in Figure 7f, provides a comprehensive understanding of their viscoelastic behavior across a therapeutically relevant temperature range (20–100 °C). Tanδ, the ratio of loss modulus to storage modulus, indicates the degree of mechanical energy dissipation during cyclic loading. Across all formulations (FP_*H*_‐FR25, FP_*M*_‐FR50, and FP_*L*_‐FR100), tanδ decreased with rising temperature, a common behavior attributed to polymer softening and reduced frictional interactions as materials approach their rubbery plateau. Notably, FP_*H*_‐FR25 exhibited the highest damping capacity, with tanδ values reaching ≈0.16 at 25 °C and remaining significantly elevated within the therapeutic window of 30–60 °C. This behavior contrasts with FP_*M*_‐FR50 and FP_*L*_‐FR100, which displayed moderate (0.065) and low (0.045) room‐temperature tanδ values, respectively. The enhanced damping in FP_*H*_‐FR25 is linked to its finer pore morphology and higher porosity, which increase interfacial friction and promote localized pore wall buckling mechanisms that dissipate energy more effectively. In contrast, the coarser pores and less uniform microstructure of FP_*L*_‐FR100 result in lower frictional losses and a more elastic response under cyclic loading.

These distinctions are not merely mechanical but have direct implications for thermo‐therapeutic functionality. The consistently higher tanδ observed in FP_*H*_‐FR25 indicates enhanced damping capacity, which is beneficial for efficient mechanical energy dissipation during cyclic loading. This property supports the material's ability to attenuate vibrations and maintain thermal comfort during dynamic use, contributing to its suitability for thermo‐therapeutic applications. This correlation between damping behavior and thermal efficacy aligns with earlier findings in Figure 7d,e, where FP_*H*_‐FR25 demonstrated slower cooling rates (*Q*_*H*_ ≈ 0.126 °C s^−1^) and superior heat retention compared to higher‐flow‐rate counterparts. Furthermore, the FP_*H*_‐FR25 maintains a relative tanδ advantage (0.12–0.14) throughout the operating temperature range, indicating stable energy dissipation and user comfort under dynamic physiological conditions. In summary, these results emphasize the importance of micro‐architectural tuning via flow rate‐controlled 3D printing to achieve customized damping and thermal responses. The synergy between mechanical compliance and spatially controlled thermal delivery, particularly in AI‐designed foams, positions these architectured materials as promising candidates for multifunctional thermo‐therapeutic platforms. Such platforms could enable personalized rehabilitation devices that integrate targeted heat application, mechanical support, and pressure point relief within a single, customizable structure, addressing a wide spectrum of therapeutic needs with enhanced efficacy.

## 3 Conclusion

This work establishes a broadly impactful and scalable platform for fabricating architected PU foams through the integration of static mixer‐enabled reactive extrusion and AI‐assisted generative design. By leveraging 3D printing, hierarchical porous structures can be rapidly prototyped with precise control over cell size (0.2–1.2 µm) and porosity (65–95%), all achieved in a single step under ambient conditions, without post‐processing. The printed foams demonstrate a rare combination of low thermal conductivity (as low as 0.067 W m^−1^ K^−1^) and outstanding mechanical resilience (over 90% elastic recovery after 5000 cycles), making them ideal for multifunctional applications. The adoption of a multi‐agent LLM framework enables rapid translation of bioinspired geometries into customizable CAD architectures, allowing AI to play a central role in accelerating the design‐to‐manufacture cycle for porous materials. This digital design capability, combined with tunable mechanical and thermal responses, empowers the creation of next‐generation devices that can be tailored for distinct application needs from impact‐absorbing protective gear and ergonomic helmets to precision‐controlled wearable thermotherapy systems. By enabling synergistic tuning of thermal insulation, energy dissipation, and ergonomic conformity, this platform offers a transformative solution for adaptive materials and device innovation across healthcare, sports, defense, and sustainability domains.

## 4 Experimental Section

### Materials

Trimethylolpropane ethoxylate (TMPe) polyol (*M*_*n*_ ≈ 1014~gmol^−1^, toluene diisocyanate (TDI, mixture of 2,4‐ and 2,6‐isomers), dibutyltin dilaurate (DBTDL, 95% purity), and fumed silica (FSi) (SiO_2_, specific surface area 395 m^2^/g, CAS #112945‐52‐5) were obtained from Sigma‐Aldrich (USA). All reagents were used as received without further purification.

### Polyurethane Mixing Ratios

The molar ratio of TMPe to TDI for complete PU formation was maintained at 1.65:1. For practical formulation and comparison, the components of the PU foam reaction—TMPe, TDI, deionized (DI) water, dibutyltin dilaurate (DBTDL) catalyst, and FSi were normalized by weight. The weight distribution of each component is summarized in Table 1. TMPe constitutes ≈72.94% of the total weight, followed by TDI at 20.8%. Water and catalyst are present in smaller amounts (1.55% and 1.88%, respectively), while FSi contributes 2.8% of the total weight. This formulation provides a consistent basis for rheological and mechanical characterization of the printed foams.

### Preparing Feedstocks

Feedstocks A and B, corresponding to Barrels 1 and 2 in the dual‐syringe setup (Figure 1b), constitute the two primary components of the polyurethane (PU) system. Each feedstock is a pre‐formulated mixture of TMPe, TDI, and process‐specific additives designed to regulate reaction kinetics and foam morphology. As summarized in Table 1, 46.06% of the total formulation weight (originating from the 72.94% TMPe fraction) is combined with DI water and dibutyltin dilaurate (DBTDL) catalyst, following their respective weight ratios, to prepare Barrel 1. This mixture is homogenized using a VWR VM 3000 analog vortex mixer to ensure uniformity, after which FSi is incorporated to achieve the desired thixotropic properties. Barrel 2 contains the remaining TMPe blended with 20.84% TDI to form the pre‐mixed TDI–TMPe component. To prevent unintended moisture interference, the TDI is pre‐dried at 60 °C under vacuum for 24 h before mixing. This two‐part formulation ensures consistent reactivity and reproducible foam properties during static mixing and DIW.

### Foaming Process

The foaming process was performed using a dual‐barrel cartridge system, which delivered the two precursor components (Table 1) into a static mixer equipped with 16 helical elements (each 22 mm in length) to ensure thorough homogenization. Upon mixing, the reactive stream simultaneously initiated foaming and PU polymerization, producing a preformed ink suitable for DIW. The process was conducted at controlled flow rates of 0.25, 0.5, and 1.0 mL min^−1^, where the residence time within the static mixer dictated the degree of precursor mixing, bubble nucleation, and initial gelation. Lower flow rates provided extended residence times and finer pore structures, while higher flow rates favored larger pores and reduced uniformity. The resulting foams were deposited directly onto the build platform, with the entire process visually monitored to ensure consistent expansion and assess the impact of flow rate on structural integrity. Key foam properties, including cell size distribution and bulk density, were recorded for subsequent analysis.

### 3D Printing

The 3D printing process employed a DIW system using preformed PU feedstock. Controlled extrusion was achieved via dual mechanical syringe pumps (KD LEGATO 200) with a flow rate accuracy of ±0.35%. The printing platform was adapted from an open‐source Creality3D Ender 3 system, providing a build volume of 220 mm × 220 mm × 300 mm along the *x*, *y*, and *z* axes, respectively, and a positional accuracy of ±0.1 mm. The reactive feedstock was delivered through a flexible feed tube to a conical plastic nozzle, enabling deposition and in situ foaming along predefined G‐code toolpaths derived from CAD or AI‐generated designs. This setup allowed precise layer‐by‐layer assembly of architected foam structures, with the dynamic foaming process captured during deposition to monitor uniformity and expansion behavior.

### Characterization

### Characterization—Fourier Transform Infrared (FTIR)

Attenuated Fourier transform infrared spectroscopy analysis was carried out using a Spectrum Two spectrometer from Perkin Elmer (Greenville, SC) by collecting absorbance readings in the infrared range of 4000 to 500 cm^−1^ for foamed samples prepared via vortex and static mixing. In contrast, a potassium bromide (KBr) FTIR spectroscopy analysis was performed for solvent samples. Each foam sample was examined with a total of 64 scans, and each solvent sample was examined with 32 scans, with a resolution of 4 cm^−1^. Spectra were collected for pure TMPe, TDI, and premixed TDI‐TMPe, which were used as solvent‐phase samples, while foamed samples included vortex‐ and static‐mixed foams.

### Characterization—Rheology

The viscosity and viscoelastic properties of the feedstock were tested using a TA instrument (i.e., Discovery Hybrid Rheometer 2, DHR 2), with an ETC stainless steel parallel plate setup with a 100 µm truncation gap and 50 µm offset trim gap at RT. Approximately 2 mL of sample volume was used to maintain uniformity across the tests, and each sample was run three times to minimize error, maintaining a geometry gap of 100 µm. The flow behavior of the material under shear was visualized by observing a change in viscosity over the shear rate ranging from 0.1 to 100 s^−1^ to simulate shear thinning behavior, a prerequisite for successful DIW. Each test was preceded by a pre‐conditioning at a shear rate of 1 rad s^−1^ for 60 s to eliminate any residual stresses and normalize test conditions. A 5 mL batch of TDI‐TMPe mixture based on Table 1 recipe was prepared without its additives (DI water and DBTDL). To this mixture, 0, 1, 2 and 3 wt% of FSi was added as a thixotropic agent and analyzed.

In conjunction, an oscillation test was performed at a constant shear rate of 10 rad s^−1^ for a duration of 300 s in samples prepared with the formulation as detailed in Table 1. A 5 mL batch of vortex‐mixed, preformed PU feedstock was prepared, and ≈2 mL was deposited for testing. Another 10 mL batch of preformed mixture was prepared as described in Table 1, and 2 mL deposited using static mixing to simulate the printing mechanism at each flow rate (i.e., 0.25, 0.5, and 1 mL min^−1^). Since the reaction is spontaneous, there were two testing conditions: tests run (a) immediately after deposition, and (b) after a 60s saturation window for the foam to rise. All tests were conducted at RT (i.e., ≈25 °C).

### Characterization—Thermogravimetric Analysis (TGA)

TA Instruments TGA 550 was used to analyze the thermal stability of the formed foams, with thermal degradation temperatures used as reference data for further tests. Each test was conducted from RT to 900 °C at a ramp rate of 10 °C min^−1^ with a sample size of ≈ 15 mg on a high‐temperature platinum (Platinum HT) pan. Samples tested were produced using vortex mixers as per Table 1, and static mixers as foaming methods as described in Table 1 at different flow rates.

### Characterization—Differential Scanning Calorimetry (DSC)

The thermal degradation data obtained from TGA were utilized as a reference to define the upper temperature limits for the DSC runs. These experiments were conducted with a TA Instruments Discovery DSC 250 instrument. Each DSC run was performed from room temperature (RT) to 210 °C with a ramp rate of 5 °C min^−1^ using a sample size of ≈ 10 mg.

### Characterization—Mechanical Testing, Dynamic Mechanical Analysis (DMA)

Tensile and compressive mechanical tests were performed using a TA Instruments Discovery Hybrid Rheometer, equipped with specific tensile and compression fixtures. All measurements were conducted at a constant loading rate of 100 µm s^−1^. Specimens were carefully loaded in the instrument to ensure repeatable gripping and alignment.

The DMA experiments were conducted on the same instrument utilizing the FCO environmental oven module, which enabled temperature ramps across a broad range. High‐temperature measurements were achieved using controlled oven heating, while sub‐ambient temperatures were reached by attaching a liquid nitrogen (LN_2_) cylinder. This setup allowed for comprehensive evaluation of viscoelastic properties and thermal transitions in the PU foams under compression, extending from cryogenic conditions to temperatures above ambient.

### Characterization—Porosity Characterization‐SEM

Foam specimens were sectioned perpendicular to the printing axis using a stainless steel razor blade to minimize mechanical distortion of the pore edges. Four cross‐sections per sample were imaged. Each sample was affixed onto aluminum stubs with conductive carbon tape and coated with a ∼5 nm gold layer using a sputter coater (Leica EM ACE600) to ensure surface conductivity. High‐resolution SEM imaging (Thermo Fisher Scientific (FEI) Teneo, field emission scanning electron microscope (FESEM), 10 kV, 10 mm working distance) was conducted at random locations across the sample, and four different samples, capturing multiple representative exposures for statistical variety.

Captured SEM micrographs were processed with ImageJ v1.53 (NIH). Raw images underwent standard grayscale normalization and histogram‐based contrast enhancement to accentuate pore boundaries. Segmentation was achieved using automated thresholding algorithms (Otsu or Li methods), delineating the polymer matrix (bright phase) from voids (dark phase). Subsequent binary conversion enabled quantification of pore domains.

For each micrograph, the “Analyze Particles” module was used to compute the areal fraction of pores, expressed as 2D porosity. At least three non‐overlapping regions per sample were analyzed and averaged over four slices of a sample to yield sample‐level data. Observed pore geometries (size, distribution) and porosity values were validated against manual annotation for consistency.

### Characterization—Porosity Characterization‐Vacuum Infiltration

Porosity was also determined gravimetrically by vacuum‐assisted water infiltration. Samples were dried at 60°C under vacuum for 24 h and weighed (*W*_*d*_). Dried samples were placed in a vacuum desiccator containing deionized water. The chamber was evacuated for 40 min to remove trapped air from pores, then vented slowly to atmospheric pressure, ensuring water infiltration. For 1 h, samples were left in the chamber to reach saturation, later removed, surface‐blotted to remove excess water, and weighed immediately (*W*_*s*_).

Porosity (ϕ) was calculated as

(1) $$\phi =\frac{\left(W_{s}-W_{d}\right)}{\rho_{\text{water}}\cdot V_{\text{sample}}}$$

where ρ_water_ is the density of water and *V*_sample_ is the geometric volume of the sample.

### Characterization—Thermal Conductivity Measurement

The thermal conductivity of the prepared PU foam samples was determined using the guarded hot plate method, which is widely recognized for its accuracy in measuring thermal properties of insulation materials. Each foam sample was measured three times to ensure repeatability and reliability of the results. The samples had rectangular dimensions (10 mm × 10mm × 3 mm) and porosity levels ranging from 65% to 95%. All measurements were conducted under carefully controlled laboratory conditions with ambient temperature maintained between 19 and 20°C and relative humidity (RH) between 25% and 30%, minimizing the influence of moisture on the results. The test setup and measurement procedure are illustrated in Figure S6 (Supporting Information).

## Conflict of Interest

The authors declare no conflicts of interest.

## Supporting information



Supporting Information

## Data Availability

The data that support the findings of this study are available from the corresponding author upon reasonable request.
