# Supplementary material for: AI‐Directed 3D Printing of Hierarchical Polyurethane Foams
Source: Adv Sci (Weinh). 2025 Nov 29;13(8):e15122. doi: 10.1002/advs.202515122 (PMC12884732; doi:10.1002/advs.202515122)
Supplement: Supplementary file 1 — Supporting Information [file ADVS-13-e15122-s001.pdf]

# Supporting Information

# AI-Directed 3D Printing of Hierarchical Polyurethane Foams

Dhanush Patil[1], Jie Tian[1], Kun Jiang[1], Clarissa Westover[2], Sri Vaishnavi Thummalapalli[1], Arunachalam Ramanathan[1], Arpita Shome[3], Natalie Crutchfield[3], M. Taylor Sobczak[1], Sean Lee[1], Hongyue Sun[1], Elizabeth J. Brisbois[3], Hitesh Handa[3,4], Timothy E. Long[5], Xianqiao Wang[6], and Kenan Song*[7]

[1]Mechanical Engineering, College of Engineering, University of Georgia, 302 E. Campus Rd, Athens, Georgia, 30602, USA
[2]School for the Engineering of Matter, Transport, and Energy and Biodesign Center for Sustainable Macromolecular Materials and Manufacturing (SM3), Arizona State University, Tempe, Arizona, 85287, USA
[3]School of Chemical, Materials & Biomedical Engineering, College of Engineering, University of Georgia, Athens, Georgia, USA 30602
[4]Department of Pharmaceutical & Biomedical Sciences, College of Pharmacy, University of Georgia, Athens, Georgia, USA 30602
[5]School of Molecular Sciences and Biodesign Center for Sustainable Macromolecular Materials and Manufacturing (SM3), Arizona State University, Tempe, Arizona, 85287, USA
[6]Professor of Mechanical Engineering, School of Environmental, Civil, Agricultural and Mechanical (ECAM), University of Georgia, Athens, GA, USA 30602
[7]Associate Professor of Mechanical Engineering, School of Environmental, Civil, Agricultural and Mechanical (ECAM) and School of Chemical, Materials, and Biomedical Engineering (CMBE), University of Georgia, Athens, GA, USA 30602

*Corresponding author: kenan.song@uga.edu

# Contents

**1 Thermal Imaging of Batch Mixing and 3DP Processes** **6**

**2 3DP Static Mixing Accessory** **7**

**3 3DP Flow Rate Effects on Foaming Behavior** **8**

**4 Flow Rate Effects on Pore Size** **9**

**5 Compressive Strength and Porous Architecture** **10**

**6 Thermal Stability** **11**

**7 Guarded hot plate method, thermal conductivity ($k$)** **12**

**8 Thermal Conductivity Simulations** **13**

# List of Figures

**S1** Thermal images illustrating the exothermic polyurethane (PU) formation reaction upon mixing polyol and isocyanate components. (a) Batch mixing via vortex agitation shows localized heating within the vial, with temperatures rising to approximately 39.0 °C relative to ambient, confirming the strongly exothermic nature of urethane bond formation. (b) Continuous static mixing during direct ink writing distributes the heat along the flow path through the silicone tubing to the nozzle exit, demonstrating more uniform thermal evolution during in situ polymerization and deposition. . . . . . . . . . . . . . . . . . . . . . . . . . . 6

**S2** Commercial static mixer used for blending two-component polyurethane (PU) foams during direct ink writing. The mixer measures 2.9 inches in length and contains 16 helical mixing elements that promote laminar splitting, shearing, and recombination of the polyol and isocyanate streams, ensuring homogeneous mixing at a fixed 1:1 ratio. Its transparent housing enables real-time visual monitoring of the mixing quality, while the internal geometry minimizes dead zones and waste, supporting continuous and uniform reactive extrusion for hierarchical foam fabrication. . . . . . . . . . . . . . . . . . . . . . . . . . . . . . . . . 7

**S3** (a) A digital image of a square bayonet static mixer purchased from Amazon. (b) The SEM of the cross-section shows a similar open-cell porous structure when mixed using a flow rate of 0.5 mL $min^1$, and (b) an FTIR spectrum comparing the vortex (V. Mixed), helical (H. Mixed), and bayonet (B. Mixed) foam samples, showing overlapping urethane linkages. To benchmark mixing designs, preliminary experiments were conducted using both a helical static mixer and a square bayonet static mixer of equivalent volume. Both geometries were found to deliver highly comparable mixing efficiencies and homogenization performance, as confirmed by FTIR and SEM analysis of resultant foams. Importantly, it was observed that the choice between square bayonet and helical geometry did not affect mixing outcome, as long as the mixer maintained a 50 mL total mixing volume and the component ratio was kept constant. Instead, differences in foam morphology were attributed directly to the flow rate applied during mixing and not to the static mixer shape itself. Accordingly, subsequent DIW printing was performed using the helical mixer. . . . . . . . . . . . . . . . . . . . . . . 7

**S4** (a) Visual demonstration of the effect of flow rate on the foaming behavior of polyurethane. The foamed region is divided by a dashed red line, where the left side (Lo-FR) corresponds to a lower flow rate ($< 50$ mL $min^{-1}$), while the right side (Hi-FR) represents a higher flow rate ($> 50$ mL $min^{-1}$). Distinct differences in foam structure and morphology are visually observed across the flow rate boundary. (b) Schematic illustration of the pathways of foam printing at different rates. The green arrow represents the direction of flow, transitioning from low (Lo) to high (Hi) flow rates across the red dashed line. This parameter study was used to systematically investigate how varying the flow rate during foam formation influences the resulting foam morphology and properties. Insights gained from this analysis guided the selection of representative samples at low, medium and high flow rates for further detailed characterization, compared in the main manuscript. . . . . . . . . . . . . . . . . . . . . . . 8

**S5** Representative SEM images illustrating the effect of flow rate on the pore structure of polyurethane (PU) foam. Images (a-e) show PU foam cross-sections produced at increasing flow rates, from low (left) to high (right), as indicated by the orange arrow. Each image displays the resulting pore morphology and size distribution at the respective flow rate. As indicated with the arrows, from left to the right corresponds to flow rates 0.25 mL min $^{-1}$; 0.5 mL min $^{-1}$; 1 mL min $^{-1}$. The scale bars shown in each image are referenced to 2 mm. 9

**S6** Compressive stress–strain curves for three representative polyurethane foams, $FP_H$-FR25, $FP_M$-FR50, and $FP_L$-FR100, tested at a constant strain rate of 100 $\mu$m $s^{-1}$ under controlled environmental conditions (relative humidity 25–30%, temperature 19–20°C). The figure illustrates stress (MPa) against strain (%) for each sample, capturing the differences in mechanical response due to processing parameters. Calculated energy absorption values (418.41, 18.43, and 2.83 J $m^{-3}$) are annotated for each corresponding curve. Above the graph, inset SEM images display the distinct pore morphologies and microstructural architecture: $FP_H$-FR25 features large, thin-walled, and highly interconnected pores; $FP_M$-FR50 shows intermediate structure; and $FP_L$-FR100 exhibits smaller, thicker-walled, and more densely packed pores. The figure demonstrates how architectural tuning through printing flow rate directly impacts the compressive performance and energy dissipation behavior of the foams. . . . . . . . . . . . . . . . . . . . . . . . . . . . . . . . . . . . . . . . 10
**S7** (a) Presents comparative thermogravimetric analysis (TGA) curves of the polyurethane foam under inert ($N_2$) and oxidative (air) atmospheres. The foam exhibits a higher onset temperature for major weight loss in $N_2$ (∼275–300 °C), with air causing earlier degradation (shifted lower by ∼20–25 °C) and more pronounced post-decomposition mass loss due to ongoing oxidation of char residues. This highlights the increased susceptibility of the foam to thermal breakdown under oxidative conditions. (b) Here, the isothermal mass retention profiles at various aging temperatures in air are shown. Across practical service temperatures (40–150 °C), the foam retains nearly all its mass during 90-minute exposures, confirming robust thermal stability and resistance to long-term degradation. Notably, incipient weight loss is first observed at 150 °C, indicating that extended exposure at this temperature could gradually impact foam integrity, whereas mass loss at 200 °C is already pronounced ($\Delta$w $\approx$ 6% after 5000 s), marking the onset of structural breakdown. Together, these results validate that PU foams are highly durable for normal thermal cycling and moderately elevated environments; however, continuous exposure above 150 °C leads to slow degradation and becomes significant only under more extreme conditions. . . . . . . . . . . . . . . . . . 11
**S8** The apparatus consists of a rectangular chamber with the central test region sandwiched between two Peltier plates (purchased from Amazon), functioning as the cold plate (top) and hot plate (bottom). Polyurethane foam (PU foam) is positioned between the plates, with thermal paste applied at the plate-foam sample interfaces to reduce thermal resistance. The entire assembly is surrounded by a thick layer of insulating material (expanded polystyrene (XPS)) to minimize heat exchange with the surrounding environment. PU foam samples are placed centrally, and their temperature profile is established by the controlled temperature difference between the hot and the cold Peltier plates using two DC power supplies. The schematic illustrates the resulting thermal gradient, with heat flowing from the hot plate upward to the cold plate. The setup was made to reduce the air gap as much as possible to reduce cross thermal transport and promote unidirectional heat transfer from the hot-cold plate, creating a defined thermal gradient. . . . . . . . . . . . . . . . . . . . . . . . . . . 12
**S9** (a-d) Mathematically modeled porosities between 15-55% to simulate thermal conductivity measurements. All models share the same domain size and boundary conditions, while porosity is introduced by randomly placing circular voids (the material is assigned by air) of varying diameters and positions. Owing to the similar temperature gradients applied, the maximum and minimum heat fluxes exhibit only slight differences, which are attributed to variations in the microstructure. Since the lower conductivity in the air, the lowest heat flux appears in the voids while the highest heat flux exists in the interface of the base and voids due to disrupted thermal pathways. This effect highlights the influence of microstructural heterogeneity on thermal transport behavior. . . . . . . . . . . . . . . . . . . . . . . . . . . . 13

**S10** (a,b) Mathematically modeled domains based on SEM cross-sections of PU foam samples with 70% porosity, constructed to simulate thermal conductivity measurements. Both models utilize identical domain sizes and boundary conditions, with porosity introduced by mapping voids (assigned as air) that replicate the actual pore geometry observed in experimental SEM images. Temperature gradients of varying magnitudes are applied to the top and bottom edges of each model, enabling a direct comparison of the resulting heat flux distributions. Color maps indicate the magnitude of local heat flux, revealing that the lowest flux occurs within the air-filled voids due to their reduced thermal pathways are more disrupted. These simulations underscore how microstructural heterogeneity, especially at high porosity, governs the spatial variation in thermal transport within PU foams. . . . . . 14

# 1 Thermal Imaging of Batch Mixing and 3DP Processes

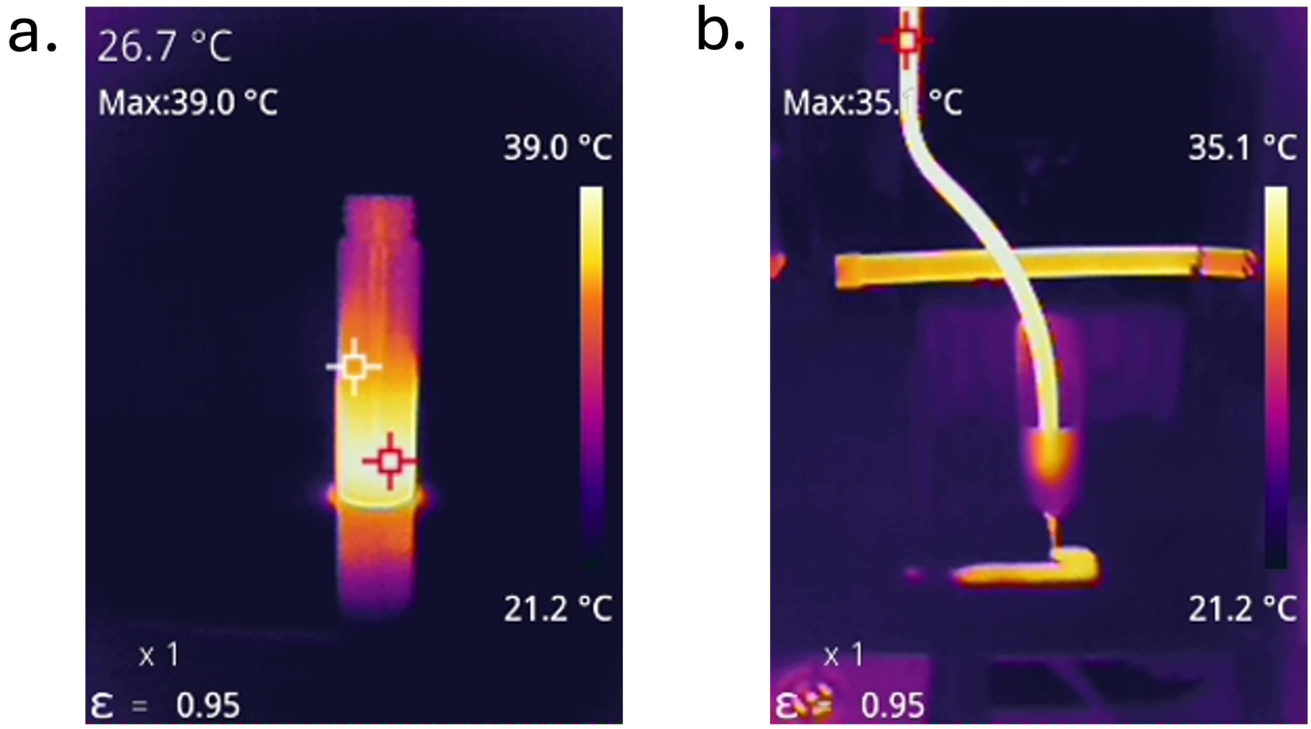


**Figure S1:** Thermal images illustrating the exothermic polyurethane (PU) formation reaction upon mixing polyol and isocyanate components. (a) Batch mixing via vortex agitation shows localized heating within the vial, with temperatures rising to approximately 39.0 °C relative to ambient, confirming the strongly exothermic nature of urethane bond formation. (b) Continuous static mixing during direct ink writing distributes the heat along the flow path through the silicone tubing to the nozzle exit, demonstrating more uniform thermal evolution during in situ polymerization and deposition.

# 2 3DP Static Mixing Accessory

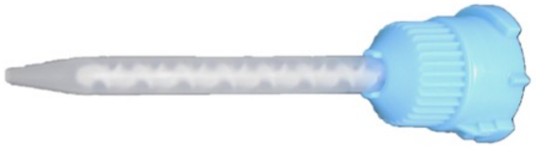

**Figure S2:** Commercial static mixer used for blending two-component polyurethane (PU) foams during direct ink writing. The mixer measures 2.9 inches in length and contains 16 helical mixing elements that promote laminar splitting, shearing, and recombination of the polyol and isocyanate streams, ensuring homogeneous mixing at a fixed 1:1 ratio. Its transparent housing enables real-time visual monitoring of the mixing quality, while the internal geometry minimizes dead zones and waste, supporting continuous and uniform reactive extrusion for hierarchical foam fabrication.

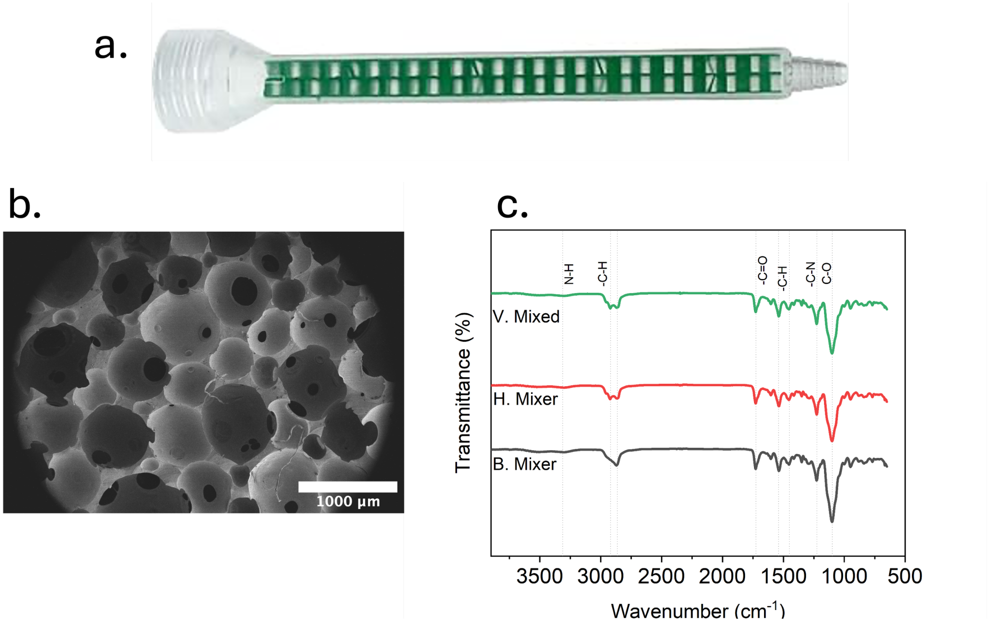


**Figure S3:** (a) A digital image of a square bayonet static mixer purchased from Amazon. (b) The SEM of the cross-section shows a similar open-cell porous structure when mixed using a flow rate of 0.5 mL min$^1$, and (b) an FTIR spectrum comparing the vortex (V. Mixed), helical (H. Mixed), and bayonet (B. Mixed) foam samples, showing overlapping urethane linkages.

To benchmark mixing designs, preliminary experiments were conducted using both a helical static mixer and a square bayonet static mixer of equivalent volume. Both geometries were found to deliver highly comparable mixing efficiencies and homogenization performance, as confirmed by FTIR and SEM analysis of resultant foams. Importantly, it was observed that the choice between square bayonet and helical geometry did not affect mixing outcome, as long as the mixer maintained a 50 mL total mixing volume and the component ratio was kept constant. Instead, differences in foam morphology were attributed directly to the flow rate applied during mixing and not to the static mixer shape itself. Accordingly, subsequent DIW printing was performed using the helical mixer.

## 3 3DP Flow Rate Effects on Foaming Behavior

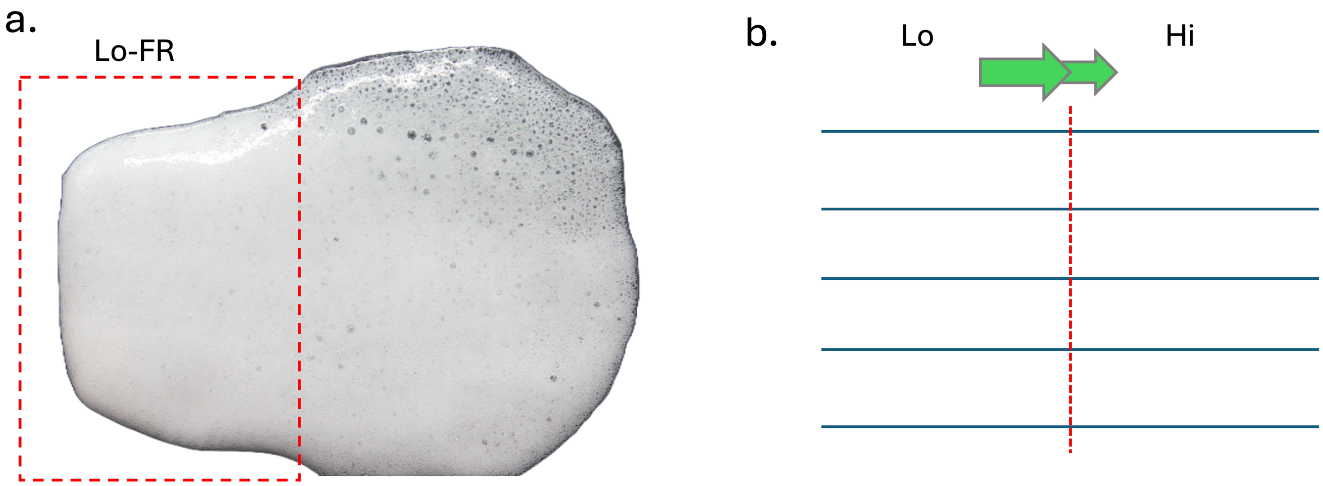


**Figure S4:** (a) Visual demonstration of the effect of flow rate on the foaming behavior of polyurethane. The foamed region is divided by a dashed red line, where the left side (Lo-FR) corresponds to a lower flow rate ($< 50$ mL min$^{-1}$), while the right side (Hi-FR) represents a higher flow rate ($> 50$ mL min$^{-1}$). Distinct differences in foam structure and morphology are visually observed across the flow rate boundary.

(b) Schematic illustration of the pathways of foam printing at different rates. The green arrow represents the direction of flow, transitioning from low (Lo) to high (Hi) flow rates across the red dashed line.

This parameter study was used to systematically investigate how varying the flow rate during foam formation influences the resulting foam morphology and properties. Insights gained from this analysis guided the selection of representative samples at low, medium and high flow rates for further detailed characterization, compared in the main manuscript.

# 4 Flow Rate Effects on Pore Size

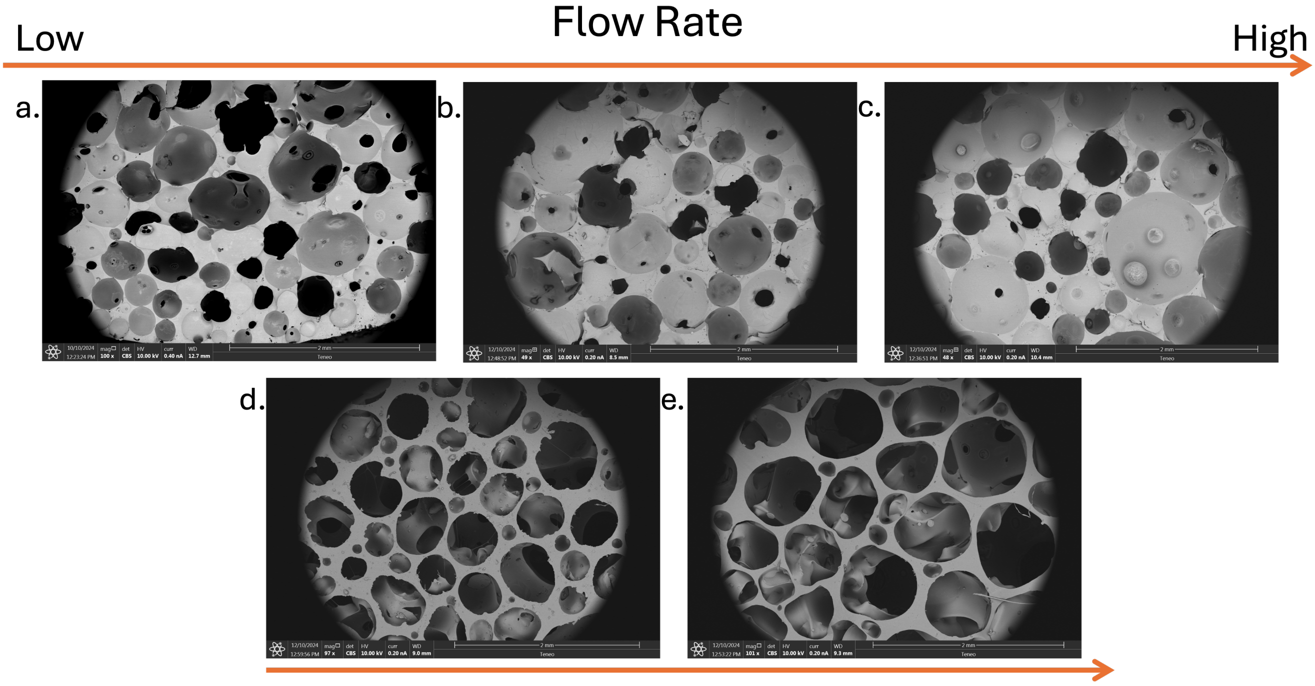


**Figure S5:** Representative SEM images illustrating the effect of flow rate on the pore structure of polyurethane (PU) foam. Images (a-e) show PU foam cross-sections produced at increasing flow rates, from low (left) to high (right), as indicated by the orange arrow. Each image displays the resulting pore morphology and size distribution at the respective flow rate. As indicated with the arrows, from left to the right corresponds to flow rates 0.25 mL min $^{-1}$; 0.5 mL min $^{-1}$; 1 mL min $^{-1}$. The scale bars shown in each image are referenced to 2 mm.

## 5 Compressive Strength and Porous Architecture

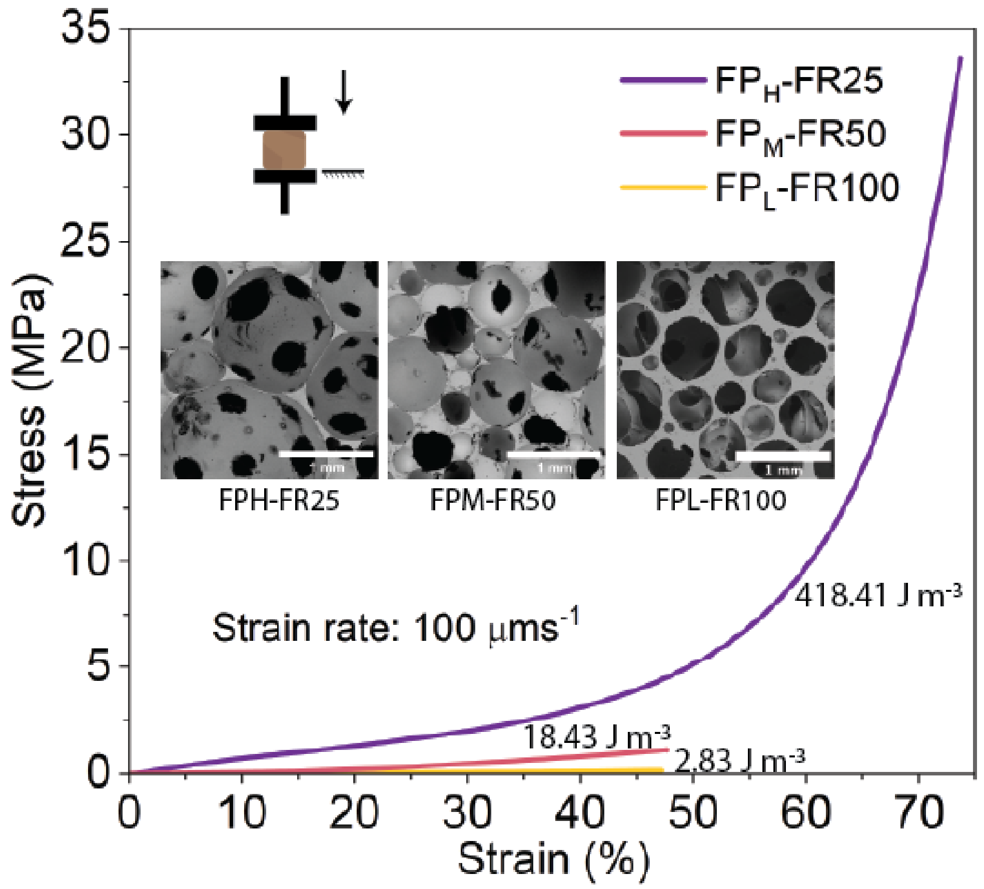


**Figure S6:** Compressive stress–strain curves for three representative polyurethane foams, $FP_H$-FR25, $FP_M$-FR50, and $FP_L$-FR100, tested at a constant strain rate of 100 $\mu$m $s^{-1}$ under controlled environmental conditions (relative humidity 25–30%, temperature 19–20°C). The figure illustrates stress (MPa) against strain (%) for each sample, capturing the differences in mechanical response due to processing parameters. Calculated energy absorption values (418.41, 18.43, and 2.83 J $m^{-3}$) are annotated for each corresponding curve. Above the graph, inset SEM images display the distinct pore morphologies and microstructural architecture: $FP_H$-FR25 features large, thin-walled, and highly interconnected pores; $FP_M$-FR50 shows intermediate structure; and $FP_L$-FR100 exhibits smaller, thicker-walled, and more densely packed pores. The figure demonstrates how architectural tuning through printing flow rate directly impacts the compressive performance and energy dissipation behavior of the foams.

## 6 Thermal Stability

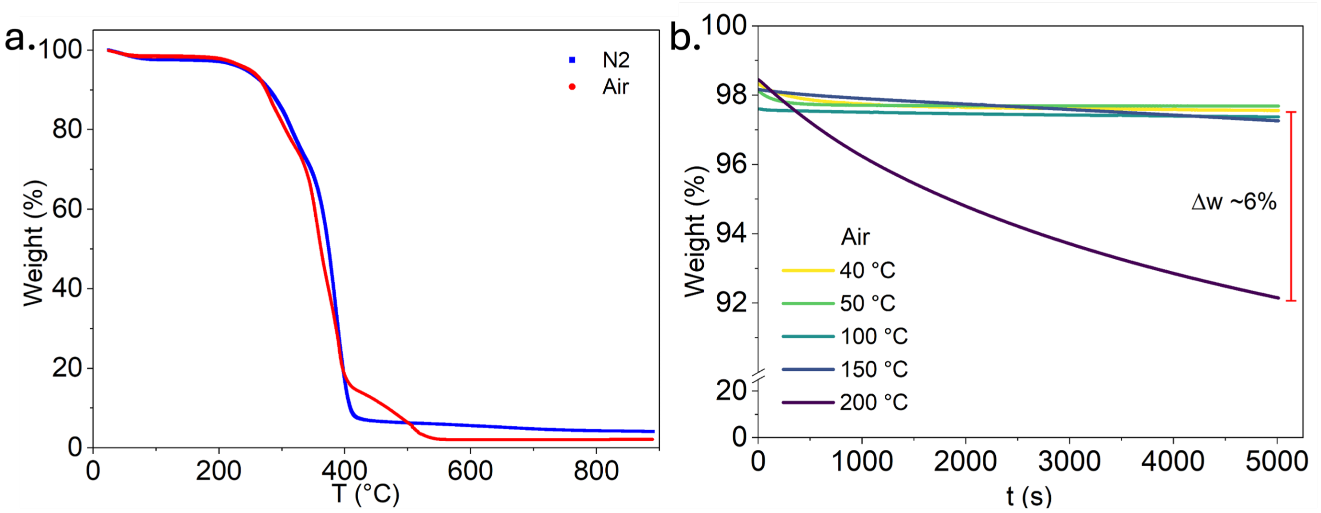


**Figure S7:** (a) Presents comparative thermogravimetric analysis (TGA) curves of the polyurethane foam under inert ($N_2$) and oxidative (air) atmospheres. The foam exhibits a higher onset temperature for major weight loss in $N_2$ (∼275–300 °C), with air causing earlier degradation (shifted lower by ∼20–25 °C) and more pronounced post-decomposition mass loss due to ongoing oxidation of char residues. This highlights the increased susceptibility of the foam to thermal breakdown under oxidative conditions. (b) Here, the isothermal mass retention profiles at various aging temperatures in air are shown. Across practical service temperatures (40–150 °C), the foam retains nearly all its mass during 90-minute exposures, confirming robust thermal stability and resistance to long-term degradation. Notably, incipient weight loss is first observed at 150 °C, indicating that extended exposure at this temperature could gradually impact foam integrity, whereas mass loss at 200 °C is already pronounced ($\Delta w \approx 6\%$ after 5000 s), marking the onset of structural breakdown. Together, these results validate that PU foams are highly durable for normal thermal cycling and moderately elevated environments; however, continuous exposure above 150 °C leads to slow degradation and becomes significant only under more extreme conditions.

# 7 Guarded hot plate method, thermal conductivity ($k$)

The guarded hot plate method was employed to measure the thermal conductivity of the polyurethane (PU) foam samples. In this custom setup, the foam specimen was sandwiched between two Peltier-controlled plates acting as the heat source (hot side) and heat sink (cold side), establishing a stable temperature gradient across the sample thickness. To minimize lateral heat losses and ensure predominantly one-dimensional heat flow, the specimen was surrounded by insulating foam and a guard ring, following established methods for polymer foam characterization.[1, 2] The temperature difference between the two plates was continuously monitored using embedded thermocouples, while the steady-state heat flux was determined from the electrical power supplied to the hot plate. The thermal conductivity was then calculated using Fourier's law, based on the measured heat flow, the applied temperature gradient, and the known sample dimensions, consistent with prior studies on low-density polymer foams and hierarchical porous systems.[3–5] Furthermore, these measurements were compared with predictions from effective medium theories, including the Hashin–Shtrikman bounds and simulation-based models, to contextualize the role of porosity and cell connectivity in tuning foam insulation performance.[6, 7]

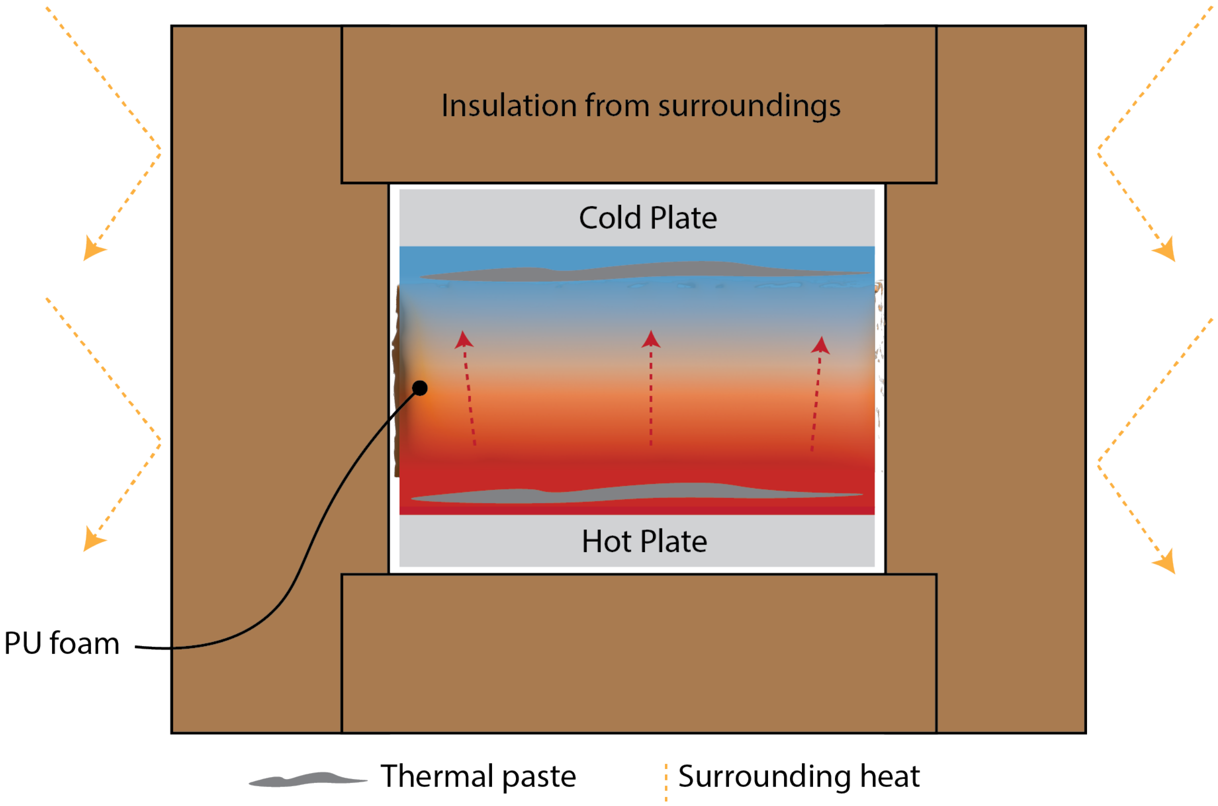


**Figure S8:** The apparatus consists of a rectangular chamber with the central test region sandwiched between two Peltier plates (purchased from Amazon), functioning as the cold plate (top) and hot plate (bottom). Polyurethane foam (PU foam) is positioned between the plates, with thermal paste applied at the plate-foam sample interfaces to reduce thermal resistance. The entire assembly is surrounded by a thick layer of insulating material (expanded polystyrene (XPS)) to minimize heat exchange with the surrounding environment.

PU foam samples are placed centrally, and their temperature profile is established by the controlled temperature difference between the hot and the cold Peltier plates using two DC power supplies. The schematic illustrates the resulting thermal gradient, with heat flowing from the hot plate upward to the cold plate. The setup was made to reduce the air gap as much as possible to reduce cross thermal transport and promote unidirectional heat transfer from the hot-cold plate, creating a defined thermal gradient.

# 8 Thermal Conductivity Simulations

To investigate the influence of porosity and thickness on thermal conductivity, heat transfer simulations were conducted using the commercial software ABAQUS. In the experiments, the temperature gradient between the heating and cooling surfaces stabilizes after a period of time, allowing heat flux to be used as an indicator of thermal conductivity. Since heat flux is directly related to the temperature gradient when material properties are constant, a steady-state analysis was used to capture the experimental behavior. Constant temperature boundary conditions were applied to the top and bottom surfaces of the sample—for example, 10 °C at the bottom and 60 °C at the top. As the heat flux distribution within the sample is not uniform, the average heat flux was calculated by integrating over the entire sample to quantify the effective thermal conductivity. To ensure consistency, the temperature gradient used for this calculation was also spatially averaged in a manner analogous to the treatment of heat flux.

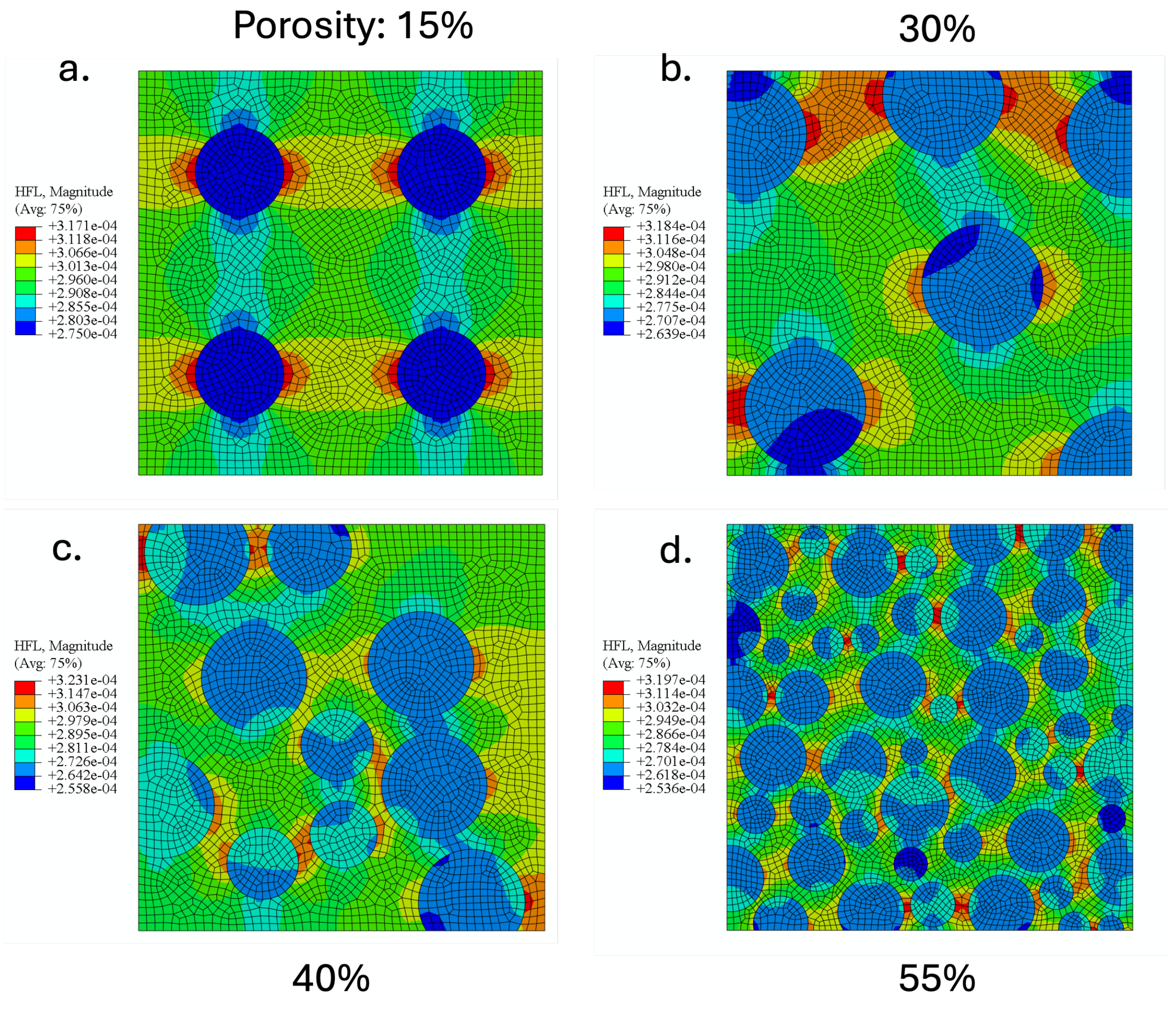


**Figure S9:** (a-d) Mathematically modeled porosities between 15-55% to simulate thermal conductivity measurements. All models share the same domain size and boundary conditions, while porosity is introduced by randomly placing circular voids (the material is assigned by air) of varying diameters and positions. Owing to the similar temperature gradients applied, the maximum and minimum heat fluxes exhibit only slight differences, which are attributed to variations in the microstructure. Since the lower conductivity in the air, the lowest heat flux appears in the voids while the highest heat flux exists in the interface of the base and voids due to disrupted thermal pathways. This effect highlights the influence of microstructural heterogeneity on thermal transport behavior.

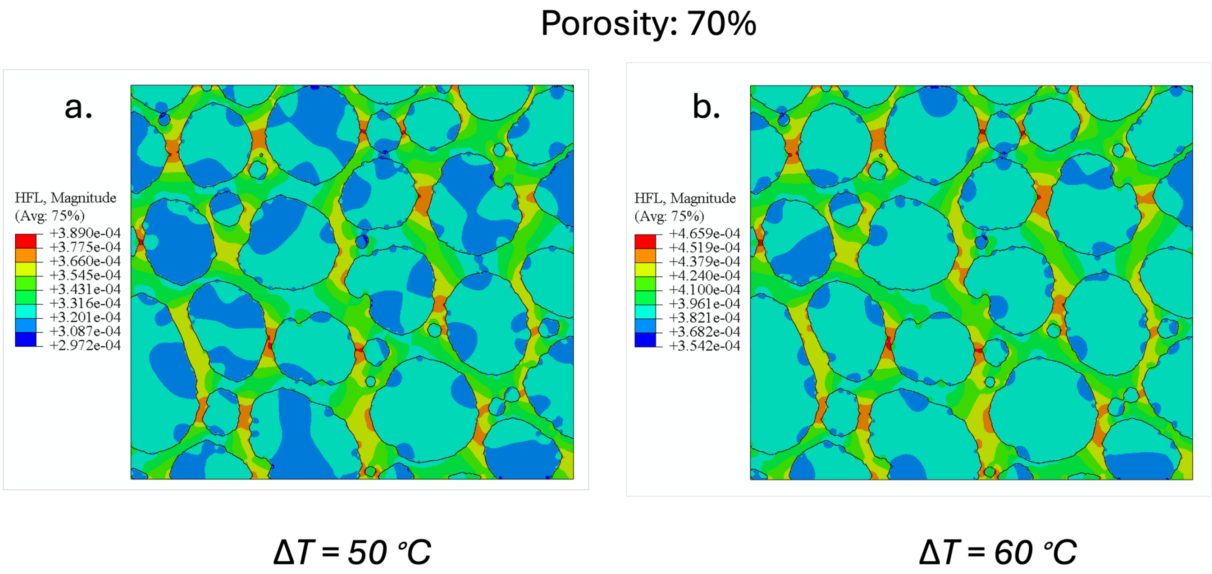


**Figure S10:** (a,b) Mathematically modeled domains based on SEM cross-sections of PU foam samples with 70% porosity, constructed to simulate thermal conductivity measurements. Both models utilize identical domain sizes and boundary conditions, with porosity introduced by mapping voids (assigned as air) that replicate the actual pore geometry observed in experimental SEM images. Temperature gradients of varying magnitudes are applied to the top and bottom edges of each model, enabling a direct comparison of the resulting heat flux distributions. Color maps indicate the magnitude of local heat flux, revealing that the lowest flux occurs within the air-filled voids due to their reduced thermal pathways are more disrupted. These simulations underscore how microstructural heterogeneity, especially at high porosity, governs the spatial variation in thermal transport within PU foams.

## References

[1] Francisco E. Berger Bioucas, Michael H. Rausch, Thomas M. Koller, and Andreas P. Fröba. Guarded Parallel-Plate Instrument for the Determination of the Thermal Conductivity of Gases, Liquids, Solids, and Heterogeneous Systems. *International Journal of Heat and Mass Transfer*, 212:124283, September 2023.

[2] Ali Rizvi, Raymond KM Chu, and Chul B Park. Scalable fabrication of thermally insulating mechanically resilient hierarchically porous polymer foams. *ACS applied materials & interfaces*, 10(44):38410–38417, 2018. Publisher: ACS Publications.

[3] Jhy-Wen Wu, Wen-Fa Sung, and Hsin-Sen Chu. Thermal conductivity of polyurethane foams. *International Journal of Heat and Mass Transfer*, 42(12):2211–2217, June 1999.

[4] Jinchuan Zhao, Guilong Wang, Chongda Wang, and Chul B. Park. Ultra-lightweight, super thermal-insulation and strong PP/CNT microcellular foams. *Composites Science and Technology*, 191:108084, May 2020.

[5] Zhanlin Shi, Xinwu Ma, Guoqun Zhao, Guilong Wang, Lei Zhang, and Bo Li. Fabrication of high porosity Nanocellular polymer foams based on PMMA/PVDF blends. *Materials & Design*, 195:109002, 2020. Publisher: Elsevier.

[6] Zvi Hashin and Shmuel Shtrikman. A variational approach to the theory of the effective magnetic permeability of multiphase materials. *Journal of applied Physics*, 33(10):3125–3131, 1962. Publisher: American Institute of Physics.

[7] Antonio L DeVera Jr and William Strieder. Upper and lower bounds on the thermal conductivity of a random, two-phase material. *The Journal of Physical Chemistry*, 81(18):1783–1790, 1977. Publisher: ACS Publications.
